# Temperature‐Dependence of the Rates of Reaction of Trifluoroacetic Acid with Criegee Intermediates

**DOI:** 10.1002/anie.201703700

**Published:** 2017-06-29

**Authors:** Rabi Chhantyal‐Pun, Max R. McGillen, Joseph M. Beames, M. Anwar H. Khan, Carl J. Percival, Dudley E. Shallcross, Andrew J. Orr‐Ewing

**Affiliations:** ^1^ School of Chemistry University of Bristol Cantock's Close Bristol BS8 1TS UK; ^2^ School of Chemistry Cardiff University Main Building, Park Place Cardiff CF10 3AT UK; ^3^ Jet Propulsion Laboratory Mail Stop 183-901 4800 Oak Grove Drive Pasadena CA 92209 USA

**Keywords:** atmospheric chemistry, Criegee biradical, kinetics, reactive intermediates, trifluoroacetic acid, zwitterions

## Abstract

The rate coefficients for gas‐phase reaction of trifluoroacetic acid (TFA) with two Criegee intermediates, formaldehyde oxide and acetone oxide, decrease with increasing temperature in the range 240–340 K. The rate coefficients k(CH_2_OO + CF_3_COOH)=(3.4±0.3)×10^−10^ cm^3^ s^−1^ and k((CH_3_)_2_COO + CF_3_COOH)=(6.1±0.2)×10^−10^ cm^3^ s^−1^ at 294 K exceed estimates for collision‐limited values, suggesting rate enhancement by capture mechanisms because of the large permanent dipole moments of the two reactants. The observed temperature dependence is attributed to competitive stabilization of a pre‐reactive complex. Fits to a model incorporating this complex formation give k [cm^3^ s^−1^]=(3.8±2.6)×10^−18^ T^2^ exp((1620±180)/T) + 2.5×10^−10^ and k [cm^3^ s^−1^]=(4.9±4.1)×10^−18^ T^2^ exp((1620±230)/T) + 5.2×10^−10^ for the CH_2_OO + CF_3_COOH and (CH_3_)_2_COO + CF_3_COOH reactions, respectively. The consequences are explored for removal of TFA from the atmosphere by reaction with biogenic Criegee intermediates.

Halogenated organic acids such as trifluoroacetic acid (TFA) form in the Earth's troposphere by oxidation of anthropogenically produced hydrofluorocarbons (HFCs), hydrochlorofluorocarbons (HCFCs) and hydrofluoro‐olefins (HFOs),^1^ and also have natural sources.^2^ They react only slowly with hydroxyl radicals and do not photolyse at actinic wavelengths.^3^ Current atmospheric models therefore incorporate surface deposition and rain‐out as their main loss processes.1b, 2 However, recent evidence from laboratory studies indicates that organic acids, and other trace atmospheric molecules, react with Criegee intermediates with room‐temperature rate coefficients that approach (or exceed) the expected gas‐kinetic limits predicted by collision rates.^4^ Barrierless reaction pathways have been identified computationally,^5^ corroborating the experimental measurements. These reactions might therefore represent a significant chemical loss mechanism for halogenated organic acids from the troposphere.

Here, we examine the temperature dependence of the reactions of CH_2_OO and (CH_3_)_2_COO with TFA, which we selected as representative of Criegee intermediate reactions with halogenated organic acids. We present rate coefficients measured over a range of temperatures spanning those encountered in the lower troposphere. Bimolecular rate coefficients were determined by the pseudo‐first‐order kinetic method for CH_2_OO + CF_3_COOH (*k*_1_), CH_2_OO + CF_3_COOD (*k*_2_) and (CH_3_)_2_COO + CF_3_COOH (*k*_3_) reactions for temperatures from 240 to 340 K and pressures from 10 to 100 torr. The measurements used cavity ring‐down spectroscopy methods described previously^6^ and in Supporting Information (SI).

Complementary quantum chemistry calculations provided energies and structures along the reaction pathways to aid interpretation of the kinetic measurements, and to guide predictions of rates of as‐yet unstudied reactions. Stationary points involved in the reactions of CH_2_OO, (CH_3_)_2_COO, *anti*‐C((*trans*‐CH_3_)=CH_2_)‐CHOO (*anti*‐methacrolein oxide) and *syn*‐CH_3_‐*trans*‐(CH=CH_2_)COO (*syn*‐methyl vinyl ketone oxide) with CF_3_COOH were calculated at the DF‐HF//DF‐LCCSD(T)‐F12a/aug‐cc‐pVTZ//B3LYP/6‐31+G(d) level of theory. The former two reactants serve as model systems, whereas the latter two were selected as possible Criegee intermediate products of the ozonolysis at each of the C=C bonds of isoprene, an important tropospheric constituent with biogenic sources.^7^ Their structures are shown in the SI. Similarities between the calculated reaction paths allow predictions of rates of reaction of TFA with the Criegee intermediates from isoprene ozonolysis which we incorporate into atmospheric chemistry models.

Figure 1 shows an example of the method for determination of *k*_2_ for the CH_2_OO + CF_3_COOD reaction. The CH_2_OO decay traces in the presence of different CF_3_COOD concentrations were fitted with a simultaneous first‐ and second‐order decay fit function:^6^

(1) $$\Delta \kappa \left(t\right)=\;\frac{k_{p}}{\frac{k_{p}}{\Delta \kappa \left(t_{0}\right)}e^{k_{p}t}-k^{{}^{'}}\left(\frac{2L}{cd}\right)+k^{{}^{'}}\left(\frac{2L}{cd}\right)e^{k_{p}t}}$$

**Figure 1:**
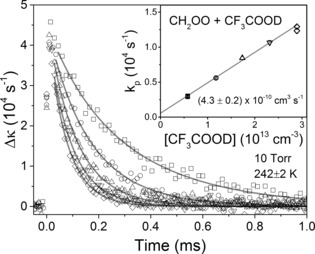
Kinetic plots for the reaction of CH_2_OO + CF_3_COOD at 10 torr total pressure and a temperature of 242±2 K. The solid lines show fits to the experimental data points obtained using Equation (1). The inset shows the pseudo‐first‐order decay rate coefficients plotted against CF_3_COOD concentration. The lowest and highest concentration measurements were repeated to ensure reproducibility. The solid line in the inset plot is a linear fit from which the bimolecular rate coefficient is obtained.

In Equation (1), $\Delta \kappa \left(t\right)$ is the change in the cavity ring‐down rate coefficient at different time delays and *k*′=*k*_obs_/*σ*_355 nm_ is the second‐order decay rate coefficient for the bimolecular self‐reaction of the Criegee intermediate scaled by its absorption cross section at a probe wavelength of 355 nm. The parameter $k_{p}$ is the rate coefficient for the TFA + Criegee intermediate reaction under pseudo‐first‐order conditions, *L* and *d* are the cavity length and the overlap length of the photolysis and probe lasers, and *c* is the speed of light. The first‐order component accounts for both unimolecular decomposition and reaction with excess CF_3_COOD. The bimolecular self‐reaction of CH_2_OO was observed to have a temperature dependence, which was included in the fitting model. The gradients of plots of *k*_p_ against CF_3_COOD concentration provide the *T*‐dependent bimolecular reaction rate coefficients, whose statistical errors varied from 1.5 to 5.7 %. Similar measurements were undertaken for the CH_2_OO + CF_3_COOH reaction. At all the temperatures studied, H/D substitution of the TFA had no significant effect on the measured rate coefficients.

Within the 10–100 torr range examined at *T*=294 K, there is no significant pressure dependence, and a rate coefficient *k*_1_(294 K)=(3.4±0.3)×10^−10^ cm^3^ s^−1^ is obtained by taking an average and 2*σ* uncertainty range of all the measurements. This rate coefficient is greater than the gas‐kinetic limiting value of 1.9×10^−10^ cm^3^ s^−1^ at 294 K calculated from collision theory using B3LYP/6‐31+G(d) optimized CH_2_OO and CF_3_COOH geometries.

We first consider the information deriving from the observed T‐dependence of the reaction rates, and then apply the resulting mechanistic understanding to further TFA reactions of atmospheric importance. We previously proposed that the self‐reactions of Criegee intermediates follow dipole capture behaviour.^8^ In the dipole capture model,^9^ the reaction cross section is greater than the physical dimensions of the reactants, and the rate coefficient *k*_d‐d_ is:

(2) $$k_{d-d}=C\sqrt{\pi /\mu }\;{{\left(\mu_{D1}\mu_{D2}\right)}^{\left(2/3\right)}\left(k_{B}T\right)}^{\left(-1/6\right)}$$

Here $\mu_{D1}$ and $\mu_{D2}$ are the dipole moments of the two reactants, μ is their reduced mass, $k_{B}$ is the Boltzmann constant, and C is a constant dependent on the anisotropy of the capture potential. Figure 2 shows a plot of the temperature dependence of the measured rate coefficients *k*_1_(*T*). This *T*‐dependence is steeper than the predictions of the dipole‐capture model obtained using Equation (2) with computed dipole moments (see SI). Similar behaviour is found for the temperature dependence of the rate coefficient *k*_3_(*T*) for the (CH_3_)_2_COO + CF_3_COOH reaction, for which the rate coefficients are approximately twice as large as for the CH_2_OO + CF_3_COOH reaction at any given *T*. For example, *k*_3_(294 K)=(6.1±0.2)×10^−10^ cm^3^ s^−1^.

**Figure 2:**
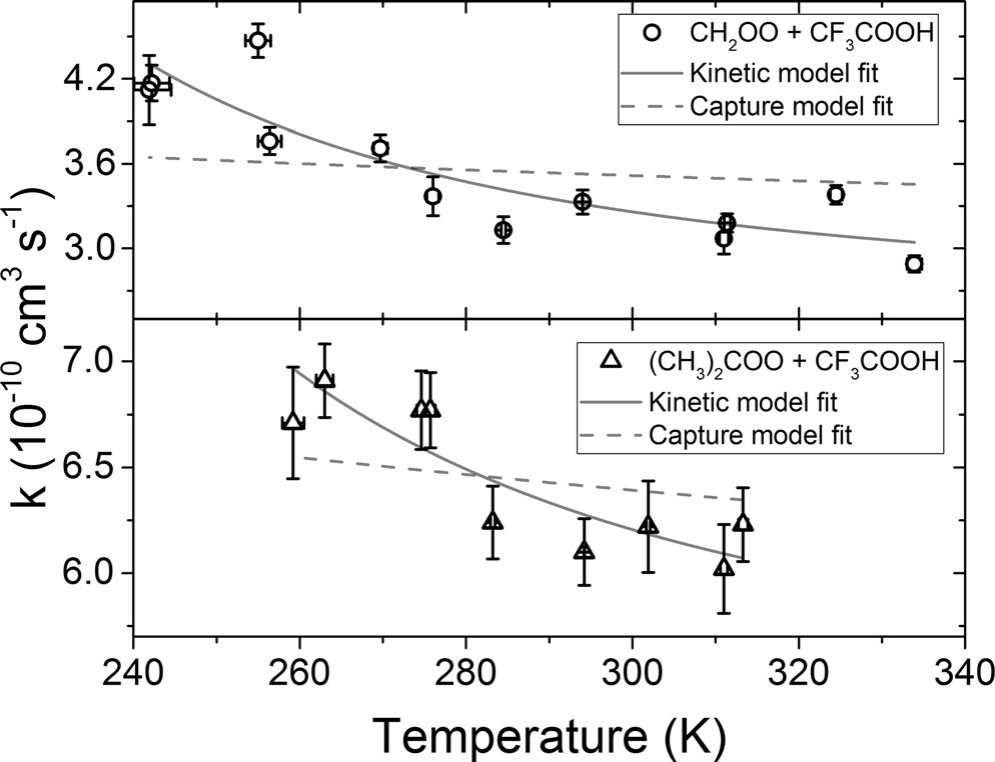
Temperature dependence of the measured rate coefficients for the CH_2_OO + CF_3_COOH and (CH_3_)_2_COO + CF_3_COOH reactions. Dashed and solid lines are fits to Equation (2) and (5), respectively.

Figure 3 shows computed energies for stationary points along the minimum energy pathways for the CH_2_OO + CF_3_COOH and (CH_3_)_2_COO + CF_3_COOH reactions. The features of both pathways are similar and we focus on the former reaction. A pre‐reactive complex coordinated by a hydrogen bond precedes a mostly entropic submerged barrier to reaction. Passage over this transition state, the properties of which are described in the SI, gives a hydroperoxy ester (HPE), CF_3_C(O)OCH_2_OOH. In this product, the H atom from TFA transfers to the CH_2_OO moiety and the carbonyl O atom of CF_3_COOH forms a bond with the C atom of CH_2_OO. This barrierless pathway is consistent with the large experimentally observed rate coefficients (Figure 2), and may account for the absence of an H/D kinetic isotope effect.

**Figure 3:**
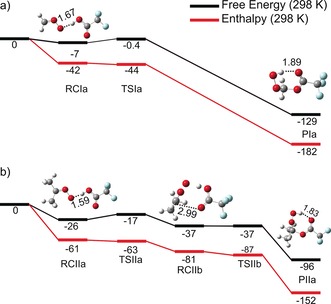
Minimum energy pathways for a) CH_2_OO + CF_3_COOH and b) (CH_3_)_2_COO + CF_3_COOH reactions, with structures and their energies calculated at the DF‐HF//DF‐LCCSD(T)‐F12a/aug‐cc‐pVTZ//B3LYP/6‐31+G(d) level of theory. Energies, given in kJ mol^−1^, are specified relative to those of the reactants (at the far left) and are shown as both reaction enthalpies and Gibbs free energies. RC, TS and P denote pre‐reactive complexes, transition states and products.

A second pathway (not shown in Figure 3) involving a different pre‐reactive complex, stabilized by dual hydrogen bonds (DHBs), is expected on the basis of prior computational studies of the CH_2_OO + HCOOH reaction.^10^ The binding energy of this DHB complex may be sufficient to influence the *T*‐dependence of the rate coefficients. Therefore, a reaction Scheme is invoked which incorporates an equilibrium between the CH_2_OO and TFA reactants and a dual hydrogen‐bonded CH_2_OO‐CF_3_COOH complex,^10^ as well as the pathway shown in Figure 3. The DHB complex has activated routes to either the HPE or a secondary ozonide (SO) product.

(3a) $$CH_{2}OO+CF_{3}COOH\;\Leftrightarrow \;DHB\;complex$$

(3b) $$CH_{2}OO+CF_{3}COOH\;\overset{}{\underset{}{\to }}HPE\;product$$

(4) $$DHB\;complex\;\overset{}{\underset{}{\to }}\;HPE\;or\;SO\;product$$

This model predicts a temperature dependence to the rate coefficient of:>

(5) $$k=A\;T^{2}exp\left(\frac{\Delta H}{RT}\right)+k_{r}$$

Here, *k*_r_ is the rate coefficient for the direct reaction (3b) (approximated to be temperature independent over the range of our study) and Δ*H*=Δ*H*_‐3a_−Δ*H*_4_ is the difference in activation enthalpies for the DHB complex to dissociate to CH_2_OO + CF_3_COOH (the reverse of (3a)) and to surmount the barrier to reaction (4). The *A*‐factor depends on the corresponding entropy changes. Equation (5) was used to fit the CH_2_OO + CF_3_COOH *T*‐dependent rate coefficients with a constrained value of the high‐temperature limit (for which *k*=*k*_r_) estimated from the data (see Figure 2). The fit returns *A*=(3.8±2.6)×10^−18^ cm^3^ s^−1^ K^−2^ and Δ*H*=13.1±1.5 kJ mol^−1^, the latter corresponding to a reaction in which the binding energy for the DHB complex is greater than the activation barrier to its reaction(s). This value is consistent with the computed enthalpy changes Δ*H*_‐3a_≈48.5 kJ mol^−1^ and Δ*H*_4_≈41 kJ mol^−1^ (at the CBS‐QB3 level) reported by Long et al. for the CH_2_OO + HCOOH reaction.^10^ A similar analysis was conducted for the (CH_3_)_2_COO + CF_3_COOH reaction, giving *A*=(4.9±4.1)×10^−18^ cm^3^ s^−1^ K^−2^ and Δ*H*=13.1±1.9 kJ mol^−1^. These fit outcomes and the corresponding entropy changes are summarized in Table S5 in the SI.

The computational methodology used for reactions of TFA with CH_2_OO and (CH_3_)_2_COO can also be applied to its reactions with Criegee intermediates from the ozonolysis of biogenic isoprene. Computed pathways for reactions of these Criegee intermediates with CF_3_COOH are found to be analogous to those in Figure 3 (see SI). The similarities indicate that the isoprene‐derived Criegee intermediate reactions (and, by extension, those of other biogenic Criegee intermediates) will approach dipole‐capture limited values and show similar T‐dependences to the CH_2_OO and (CH_3_)_2_COO + CF_3_COOH reactions. These deductions allow us to predict the loss rate of TFA in the atmosphere by reaction with the most tropospherically abundant Criegee intermediates.

Figure 4 shows computed global CF_3_COOH loss rates from reactions with Criegee intermediates, as a percentage of the overall TFA loss rate. The SI provides details of the STOCHEM‐CRI global atmospheric model and Criegee intermediate field calculations (incorporating known production and loss mechanisms) used for these computer simulations. The outcomes suggest that rapid reactions with Criegee intermediates are the dominant sink for tropospheric TFA in forested regions around the world, and that the TFA atmospheric lifetime might be as short as 4 days. Reactions of TFA with Criegee intermediates can form adducts with high O:C ratios and low vapour pressures, which encourages condensation to secondary organic aerosol (SOA). Competition between SOA formation, solar photodissociation, and adduct hydrolysis will then have consequences for the distribution of TFA and other halogenated organic acids in the environment.

**Figure 4:**
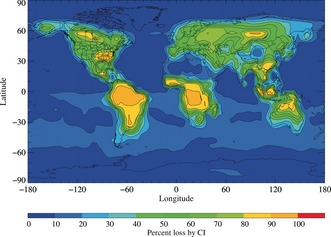
Annual mean CF_3_COOH loss contribution by Criegee intermediates (CI) using *k*_CH2OO+CF3COOH_ values for all CIs. Note: Percent loss by CI=(loss by CI×100)/(loss by CI + loss by OH + loss by deposition).

All experimental data are archived in the University of Bristol's Research Data Storage Facility (DOI: https://doi.org/10.5523/bris.1oj4r5l6s1t7k2r7oi0ekamxti).

## Conflict of interest

The authors declare no conflict of interest.

## Supporting information

As a service to our authors and readers, this journal provides supporting information supplied by the authors. Such materials are peer reviewed and may be re‐organized for online delivery, but are not copy‐edited or typeset. Technical support issues arising from supporting information (other than missing files) should be addressed to the authors.

Supplementary
