# Supplementary material for: Temperature‐Dependence of the Rates of Reaction of Trifluoroacetic Acid with Criegee Intermediates
Source: Angew Chem Int Ed Engl. 2017 Jun 29;56(31):9044–7. doi: 10.1002/anie.201703700 (PMC5575497; doi:10.1002/anie.201703700)
Supplement: Supplementary file 1 — Supplementary [file ANIE-56-9044-s001.pdf]

Angewandte Chemie
Eine Zeitschrift der Gesellschaft Deutscher Chemiker

Supporting Information

Temperature-Dependence of the Rates of Reaction of Trifluoroacetic Acid with Criegee Intermediates

Rabi Chhantyal-Pun, Max R. McGillen, Joseph M. Beames, M. Anwar H. Khan, Carl J. Percival, Dudley E. Shallcross, and Andrew J. Orr-Ewing**

anie_201703700_sm_miscellaneous_information.pdf

Table of Contents **Page**

S1. Experimental details **2**

S1.1 Production and detection of Criegee Intermediates

S1.2 Instrumental parameters and uncertainties

S1.2.1 Laser overlap length

S1.2.2 Temperature uncertainty

S1.2.3 Gas flow conditions and uncertainties

S2. Pressure dependence of rate coefficients **4**

S3. Temperature dependence of rate coefficients **4**

S3.1 Temperature dependence of background decay rate coefficients

S3.2 Temperature dependence of the capture and collision limited rate coefficients

S3.3 Temperature dependence due to reaction complex stabilization

S3.4 Representative pseudo-first-order plots

S4. Quantum Chemistry Calculations **12**

S4.1 Criegee intermediate structures

S4.2 Reaction paths for isoprene derived Criegee intermediates with CF_3COOH

S4.3 Intrinsic Reaction Coordinate profiles

S5. Atmospheric chemistry modelling **31**

S1 Experimental details

S1.1 Production of Criegee Intermediates and measurement of their temperature-dependent reaction rates

Criegee intermediates were produced by the method of Taatjes and coworkers in which an alkyl diiodide compound (either CH_2I_2 or $(CH_3)_2CI_2$ in the current work) is photolysed by an ultraviolet laser pulse (here, 355-nm wavelength) in the presence of excess molecular oxygen.[1] The concentration of the Criegee intermediate was monitored by cavity ring-down spectroscopy (CRDS) using a probe wavelength of 355 nm that overlaps the strong and broad $\tilde{B}\ {}^1A' \leftarrow \tilde{X}\ {}^1A'$ electronic absorption bands of these species. The kinetics of removal of Criegee intermediates by unimolecular and bimolecular reactions were measured by changing the time delay between the two laser pulses. The measurements used laser systems and a modified version of a flow reactor that have been described in detail previously.[2] The modified flow reactor consisted of a double-jacketed glass apparatus combined with an optical cavity for CRDS. The temperature of the flowing gas mixture was regulated by circulating a refrigerant fluid through the inner jacket using a cooling / heating unit (Huber Unistat 360). The outer jacket was sealed with dry air to provide thermal insulation. CH_2OO and $(CH_3)_2COO$ concentrations of around 2×10^{12} molecule cm^{-3}, deduced using previously published absorption cross sections,[3] and CF_3COOH concentrations of 1–3 × 10^{13} molecule cm^{-3} were used for the kinetic measurements. The overall instrumental uncertainty for CF_3COOH concentration measurement varied from 1.2 to 1.6% within the pressure and temperature range studied (see Section 1.2). The instrumental errors were combined with kinetic model fit statistical errors to give the best uncertainty estimates for the measured reaction rate coefficient. The reactor was purged with CF_3COOD overnight to reduce facile exchange of D to H during CH_2OO + CF_3OOD reaction measurements.

S1.2 Instrumental parameters and uncertainties

S1.2.1 Laser overlap length

The intersection angle of 3.8° of the photolysis and probe laser beams gave an interaction region of length 7.6 cm that was much shorter than the column of reactive gases in the flow reactor. This column length was determined by the total pressure in the reactor (which was set by regulating the flow of excess N_2) and the use of dry nitrogen purges in front of the cavity mirrors to protect them from deposition of reagents or products. The short kinetic times (<10 ms) resulting from our choice of reaction conditions ensured that first-order losses of reaction products by net flow out of the probe volume were negligible.

S1.2.2 Temperature uncertainty

The flowing gas was pre-cooled or warmed by passing it through a ~10-cm long injection arm surrounded by the fluid. The gas temperature was measured at both the points of entry and exit from the flow reactor by calibrated thermocouples, which revealed slight temperature gradients, which varied from 0 to 2.6 K over the temperature range studied. We incorporate $\pm$ 2 K typical uncertainties in temperature in the data analysis. The K-type thermocouple temperature measurement is accurate to 1 K within the temperature range studied. The data analysis does not make any corrections for changes to the absorption cross sections of the Criegee intermediates at 355 nm with temperature. However, the broad absorption bands are indicative of excitation to a dissociative excited state and the peak of the absorption band used in our study is not expected to be sensitive to changes in temperature.[3a, 4]

S1.2.3 Gas flow conditions and uncertainties

Gas flows were set using calibrated mass flow controllers (MKS 1479A and 1179A) to regulate the concentrations of alkyl diiodide precursor (diluted in excess N_2), O_2, TFA co-reactant (diluted in N_2) and N_2 bath gas. High purity N_2 and O_2 gases were obtained from Air Liquide. The precursor molecule, CH_2I_2 (99%), and reactants CF_3COOH (99%) and CF_3COOD (99%) were obtained from Sigma Aldrich and were further purified using freeze-pump-thaw cycles before use. The precursor molecule $(CH_3)_2CI_2$ was synthesized using the procedure described in our previous publication.[3b]

Mixtures of the diiodide or CF_3COOH/D diluted in N_2 were prepared and stored in 10 L Pyrex bulbs prior to their introduction into the flow reactor. The mole fractions were typically 0.1% for both diiodide and acid samples, and were determined using calibrated Ceravac manometers (Oerlikon). Several dilute bulbs were prepared for each sample, with no significant effect on rate coefficient determinations observed using the different bulbs. We estimate that bulb preparations are reproducible to 1% or better. Diiodide samples are photosensitive and were therefore stored in opaque bulbs. No dark losses were observed for acid samples or CH_2I_2, but storage of $(CH_3)_2CI_2$ led to significant losses, and these bulbs were therefore prepared 24 hours or less in advance of use. The quoted uncertainty for the Ceravac manometer pressure measurement is 0.2 %, and the calibrated MKS mass flow controller gas flow rate measurement is precise to 1 %.

S2 Pressure dependence of rate coefficients

Figure S2 shows the rate coefficients k_1 for the CH_2OO + CF_3COOH reaction measured at different total pressures and a fixed temperature of 294 K. The pressure in the flow reactor was increased by changing the flow rate of N_2 while keeping the flows of all other gases constant. Within the 10–100 Torr range examined, there is no significant pressure dependence. A pressure independent rate coefficient value of k_1(294 K) = (3.4 ± 0.3) × 10^{-10} cm^3 s^{-1} was obtained by taking an average and 2σ uncertainty range of the measurements at different pressures. The reaction is either in the high pressure limit, or is not collisionally activated and hence has negligible pressure dependence over the measurement range. These measurements were made using a previously described, room-temperature flow reactor. A pressure-independent second order CH_2OO background loss rate of 6.72 × 10^6 cm s^{-1} (corresponding to the second order rate coefficient scaled by the Criegee intermediate absorption cross section at 355 nm) was used for the simultaneous first and second order decay rate fitting.[2]

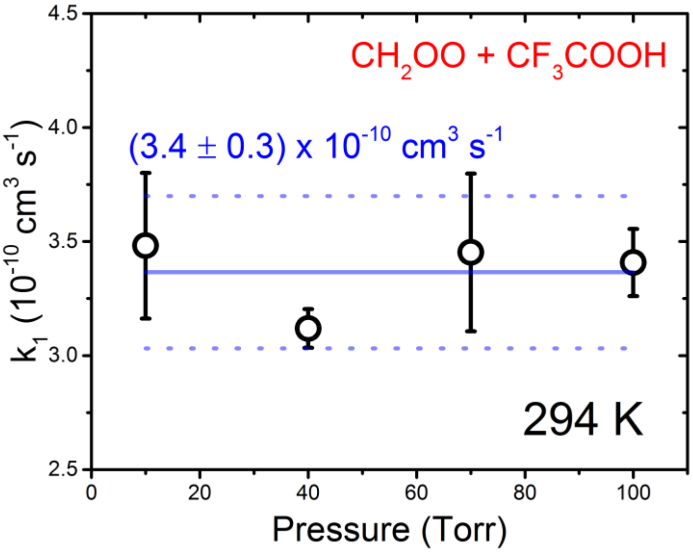

Figure S2: Pressure dependence of the rate coefficient for the CH_2OO + CF_3COOH reaction at 294 K.

S3 Temperature dependence of rate coefficients

S3.1 Temperature dependence of background decay rate coefficients

Second-order CH_2OO decay rate coefficients were measured at various temperatures. A detailed description of these measurements will be presented elsewhere. The bimolecular rate coefficient

values were found to decrease with increasing temperature and an empirical natural log fit was performed as shown in Figure S3. The fit expression was then used to obtain the background loss rate coefficient at various temperatures used for the CH_2OO + CF_3COOH reaction studies. Figure S3 also shows the temperature dependence of the second order rate coefficient for $(CH_3)_2COO$ background loss at various temperature. These values are temperature independent, and an average value was used. These second order rate coefficients were used to constrain the simultaneous first and second order fits, and this process provided effective subtraction of the effects of the Criegee intermediate self-reaction.

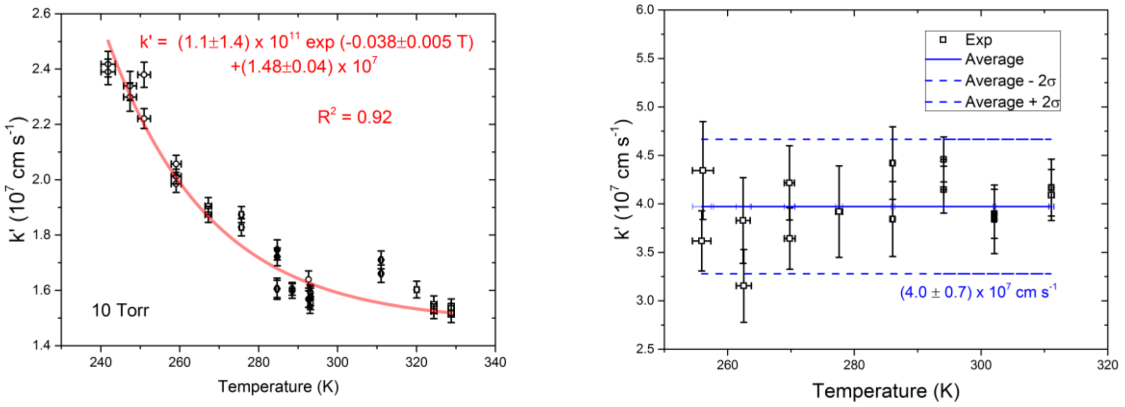

Figure S3: Temperature dependence of the rate coefficients corresponding to the Criegee intermediate self reactions. Left: CH_2OO. Right: $(CH_3)_2COO$. The second order rate coefficients have been scaled by absorption cross sections at 355 nm.

S3.2 Temperature dependence of the capture and collision limited rate coefficients

We previously proposed that the self-reactions of Criegee intermediates at room temperature follow dipole capture behaviour. In the dipole capture model, the reaction cross section is greater than the physical dimensions of the reactants because of long-range dipole-dipole attraction. The limiting dipole-capture rate coefficient $k_{d\text{-}d}$ (in CGS units) is given by Eq. (2) in the main article.

The value of C is 4.08 for an isotropic potential. Previous work of Troe and co-workers showed that in the case of adiabatic and non-adiabatic limits for an anisotropic dipole-dipole potential between two point dipoles, respective C values of 2.68 and 1.953 are obtained.[5] Dipole moment values computed at the CCSD/6-31+G(d)//B3LYP/6-31+G(d) level of theory and shown in Table S1 were used to calculate capture-limited rate coefficients from Eq. (2) at different temperatures; the values obtained exceed the experimental measurements, as is shown by comparisons presented in Tables S2 and S3.

Table S1: Dipole moment values for different reactants calculated at the CCSD/6-31+G(d)//B3LYP/6-31+G(d) level of theory.

Molecule	CF_3COOH	CH_2OO	$(CH_3)_2COO$	syn-CH_3-trans-C(CH=CH_2)CHOO	anti-C((trans-CH_3)=CH_2)-CHOO
Dipole moment (D)	2.40	5.99	6.77	7.50	7.45

Table S2: Comparison of temperature dependent rate coefficients for the CH_2OO + CF_3COOH reaction obtained from a collision theory model, the dipole-capture model (in isotropic, adiabatic and non-adiabatic anisotropic limits) and experiment. All measurements were made at 10 Torr total pressure.

Temperature (*K*)	*k* (10^{-10} cm^3 s^{-1})				
	Collision limit	Dipole-capture model			Experiment
		Isotropic	Adiabatic anisotropic	Non-adiabatic anisotropic	
241.9±2.6	1.73	10.21	6.71	4.89	4.12±0.25
242.2±2.1	1.73	10.21	6.71	4.89	4.17±0.13
255.0±1.5	1.78	10.12	6.65	4.84	4.47±0.12
256.4±1.4	1.78	10.11	6.64	4.84	3.76±0.10
269.7±0.8	1.83	10.02	6.59	4.80	3.71±0.10
276.0±0.7	1.85	9.99	6.56	4.78	3.37±0.14
284.5±0.3	1.88	9.94	6.53	4.76	3.13±0.10
294.0	1.91	9.88	6.49	4.73	3.33±0.09
311.0±0.5	1.97	9.79	6.43	4.69	3.07±0.11
311.4±0.4	1.97	9.79	6.43	4.69	3.18±0.07
324.5±0.7	2.01	9.72	6.39	4.65	3.38±0.07
333.9±0.9	2.04	9.67	6.36	4.63	2.89±0.06

For the data presented in Table S2, the collision limit rate coefficient value was obtained by using

$$k = \pi(r_{Criegee} + r_{CF_3COOH})^2 \left(\frac{8k_BT}{\pi\mu}\right)^{1/2} \quad \text{(S1)}$$

where $r_{Criegee}$ and r_{CF_3COOH} are approximate covalent radii of the Criegee intermediate and CF_3COOH obtained from B3LYP/6-31+G(d) calculations. k_B is the Boltzmann constant, *T* is temperature, and μ is the reduced mass of the reactants.

Table S3: Comparison of temperature dependent rate coefficients for the $(CH_3)_2COO + CF_3COOH$ reaction obtained from a collision theory model, the dipole-capture model (in isotropic, adiabatic and non-adiabatic anisotropic limits) and experiment. All measurements were made at 10 Torr total pressure.

Temperature	k (10^{-10} cm^3 s^{-1})				
	Collision limit	Dipole-capture model			Experiment
		Isotropic	Adiabatic anisotropic	Non-adiabatic anisotropic	
259.2±1.3	1.99	9.36	6.15	4.48	6.71±0.26
263.0±1.0	2.01	9.34	6.14	4.47	6.91±0.17
274.6±0.5	2.05	9.27	6.09	4.44	6.77±0.19
275.7±0.6	2.06	9.27	6.09	4.44	6.77±0.18
283.2±0.3	2.08	9.22	6.06	4.42	6.24±0.17
294.2	2.12	9.17	6.02	4.39	6.10±0.16
301.9±0.2	2.15	9.13	6.00	4.37	6.22±0.22
311.0±0.4	2.18	9.08	5.97	4.35	6.02±0.21
313.3±0.4	2.19	9.07	5.96	4.34	6.23±0.17

Figures S4 and S5 show the rate coefficients for the reactions of CH_2OO and $(CH_3)_2COO$ with CF_3COOH measured at different temperatures, plotted in the form suggested by Eq. (2). An approximately linear dependence is obtained, but the experimentally observed temperature dependence is steeper than that predicted by the capture model and has a negative intercept. Quantitative discrepancies between the model and the measurements indicate a reaction mechanism that is more complicated than the dipole capture model allows.

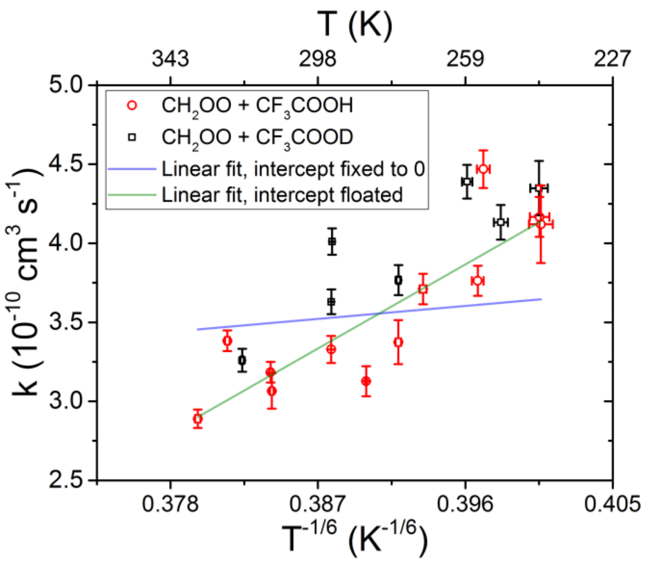

Figure S4: Temperature dependence of the rate coefficients for the $CH_2OO + CF_3COOH$ and $CH_2OO + CF_3COOD$ reactions at a pressure of 10 Torr. The solid blue and green lines show linear fits to the $CH_2OO + CF_3COOH$ dataset with the intercept values fixed to zero or allowed to vary, respectively. Linear fit expression obtained are: k [cm^3 s^{-1}] = $(9.1 \pm 0.3) \times 10^{-10}$ $T^{-1/6}$ and k [cm^3 s^{-1}] = $(6 \pm 1) \times 10^{-9}$ $T^{-1/6}$ – $(2.0 \pm 0.4) \times 10^{-9}$.

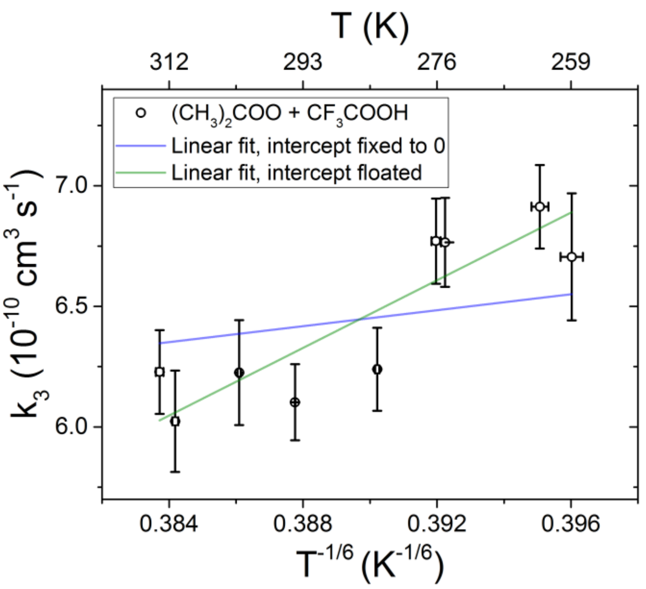

Figure S5: Temperature dependent rate coefficients for the $(CH_3)_2COO + CF_3COOH$ reaction at a pressure of 10 Torr. The solid blue and green lines show linear fits to the $(CH_3)_2COO + CF_3COOH$ dataset with the intercept values fixed to zero or allowed to vary, respectively. Linear fit expressions obtained are: k [$cm^3\ s^{-1}$] = (1.65 ± 0.02) × 10^{-9} $T^{-1/6}$ and k [$cm^3\ s^{-1}$] = (7 ± 2) × 10^{-9} $T^{-1/6}$ – (2.1 ± 0.6) × 10^{-10}.

Figure S4 also compares the rate coefficients for the CH_2OO with CF_3COOH reaction with those for the $CH_2OO + CF_3COOD$ reaction (k_1 and k_2 respectively) measured at different temperatures, and shows there to be little or no kinetic isotope effect for H/D substitution on the carboxyl group. The measured rate coefficients for the $CH_2OO + CF_3COOD$ reaction are listed in Table S4.

Table S4: Temperature dependent rate coefficients for the $CH_2OO + CF_3COOD$ reaction. All measurements were made at 10 Torr total pressure.

Temperature	k (10^{-10} $cm^3\ s^{-1}$)
242.3±1.9	4.35±0.17
251.0±1.6	4.13±0.11
258.9±1.2	4.39±0.11
276.0±0.5	3.77±0.10
293.7±0.1	4.01±0.08
293.94±0.04	3.63±0.08
320.0±0.6	3.26±0.07

The measured rate coefficients for $CH_2OO + CF_3COOH$ and $(CH_3)_2COO + CF_3COOH$ reactions at the lower temperatures used in this work are greater than those predicted by the dipole capture model.

Using Eq. (2) and the slope values from fits with intercepts allowed to vary, C values of 3.5 and 4.5 were obtained for the CH_2OO + CF_3COOH and $(CH_3)_2COO$ + CF_3COOH reactions, respectively. These values are close to the C value arising from the isotropic capture model.[5] However, the negative intercept does not have any physical meaning. In contrast, the fitted slope values with intercepts fixed at zero result in C values of 0.54 and 1.06 for the CH_2OO + CF_3COOH and $(CH_3)_2COO$ + CF_3COOH reactions, respectively. These values are lower than the C values expected for both isotropic and anisotropic capture models. The C values for the anisotropic capture model were derived for attraction between two point dipoles and may differ for structured polyatomic molecules.

S3.3 Temperature dependence due to reaction complex stabilization

The temperature dependence of the CH_2OO + CF_3COOH reaction cannot be fully explained by the dipole-capture model. The reaction channel leading to formation of the adduct product does not have significant energetic barrier, and the small entropic barrier is not expected to cause the observed temperature dependence. Instead, a reaction model with pre-equilibrium was used to quantify the observed temperature dependence of the CH_2OO + CF_3COOH rate coefficient. This model is summarized in equations (3) and (4) in the main text and is based on prior demonstration of two pre-reactive complexes in the CH_2OO + HCOOH and $(CH_3)_2COO$ + HCOOH reactions.[6] In both reactions, one of these complexes is held together by a single hydrogen bond (SHB) and can rotate over a low barrier to a reactive configuration accessing the hydroperoxy ester (HPE) product observed previously. In the CH_2OO + HCOOH reaction, the other complex is more strongly associated by double hydrogen bonds (DHBs) which hinder rotation to a geometry which reacts to the HPE or a secondary ozonide (SO) product. In the case of the $(CH_3)_2COO$ + HCOOH reaction, the DHB complex facilitates isomerization of the Criegee intermediate to vinyl hydroperoxide. The mechanism of reaction of CF_3COOH and HCOOH is expected to show similar behaviour, and the reaction scheme is given by Eqs. (3) – (4) of the main article.

Assuming steady state behaviour for the complex, the observed bimolecular rate coefficient for the loss of CH_2OO is:

$$k_{obs} = \frac{k_{3a}k_4 + k_{-3a}k_{3b} + k_{3b}k_4}{k_{-3a} + k_4} \qquad \text{(S2)}$$

The reverse reaction (–3a) of the complexation step is expected to be faster than reaction (4) because of the greater increase in entropy in the dissociation process ($k_{-3a} \gg k_4$). Thus, equation (S2) reduces to

$$k_{obs} = K_{eq}k_4 + k_{3b} \quad \text{(S3)}$$

Here $K_{eq} = k_{3a}/k_{-3a}$ is the equilibrium constant for reaction (3a). Statistical thermodynamic principles and transition state theory provide expressions for K_{eq} and k_4:

$$K_{eq} = \frac{R'T}{N_A} exp\left(\frac{\Delta S_{3a}}{R}\right) exp\left(\frac{-\Delta H_{3a}}{RT}\right) \quad \text{(S4)}$$

$$k_4 = \frac{k_B T}{h} exp\left(\frac{\Delta S_4}{R}\right) exp\left(\frac{-\Delta H_4}{RT}\right) \quad \text{(S5)}$$

Here, R' and R are the molar gas constant in different units (82.1 cm^3 atm mol^{-1} K^{-1} and 8.31 J mol^{-1} K^{-1}), N_A is Avogadro's number, k_B is the Boltzmann constant and T is the absolute temperature. ΔS_{3a} and ΔH_{3a} are the entropy and enthalpy changes for the forward reaction (3a), and ΔS_4 and ΔH_4 are the corresponding changes for activation of the complex to the transition state for product formation via (4). Equation (S3) can therefore be re-written as:

$$k_{obs} = A\,T^2 exp\left(\frac{\Delta H}{RT}\right) + k_{3b} \quad \text{(S6)}$$

Here, A and ΔH are temperature independent constant:

$$A = \frac{R' k_B}{N_A h} exp\left(\frac{\Delta S_{3a} + \Delta S_4}{R}\right) \quad \text{(S7)}$$

$$\Delta H = -(\Delta H_{3a} + \Delta H_4) = \Delta H_{-3a} - \Delta H_4 \quad \text{(S8)}$$

Equation (S8) identifies ΔH as the difference in activation enthalpies for the DHB complex to dissociate to CH_2OO + CF_3COOH (the reverse of (3a)) and to surmount the transition state barrier to reaction (4).

Equation (S6) was used to fit the CH_2OO + CF_3COOH and $(CH_3)_2COO$ + CF_3COOH rate coefficients obtained at different temperatures, as shown in the main article. Adjusted R^2 value of 0.69 and 0.67 was obtained for the CH_2OO + CF_3COOH and $(CH_3)_2COO$ + CF_3COOH reaction rate coefficients respectively. The parameters in the fits were highly correlated. Hence, for the purpose of providing a parameterization of the temperature-dependent rate coefficients across the temperature range studied, the $k_{3b} = k_r$ direct reaction rate coefficient value was fixed at values of 2.5×10^{-10} cm^3 s^{-1} (CH_2OO + TFA reaction) and 5.2×10^{-10} cm^3 s^{-1} ($(CH_3)_2COO$ + TFA reaction) and the fits to the temperature-dependent terms were repeated, giving the outcomes reported in Table S3.

Figure S5 examines the sensitivity of the kinetic model fit to the different parameters. This test shows that the model is particularly sensitive to the ΔH values.

Table S5: Thermodynamic and kinetic parameters for reactions of two Criegee intermediates with TFA. The scheme for the reaction is summarized in Eqs (3) –(4) of the main text for the case of the CH_2OO + TFA reaction. The parameters are defined in the main text.

Reaction	A (cm^3 s^{-1} K^{-2})	ΔH (kJ mol^{-1})	ΔS (J mol^{-1} K^{-1})	k_{3b} (cm^3 s^{-1})
$CH_2OO + CF_3COOH$	$(3.8 \pm 2.6) \times 10^{-18}$	13.5 ± 1.5	$-112^{+4.3}_{-9.4}$	2.5×10^{-10}
$(CH_3)_2COO + CF_3COOH$	$(4.9 \pm 4.1) \times 10^{-18}$	13.5 ± 1.9	$-110^{+5.0}_{-14.8}$	5.2×10^{-10}

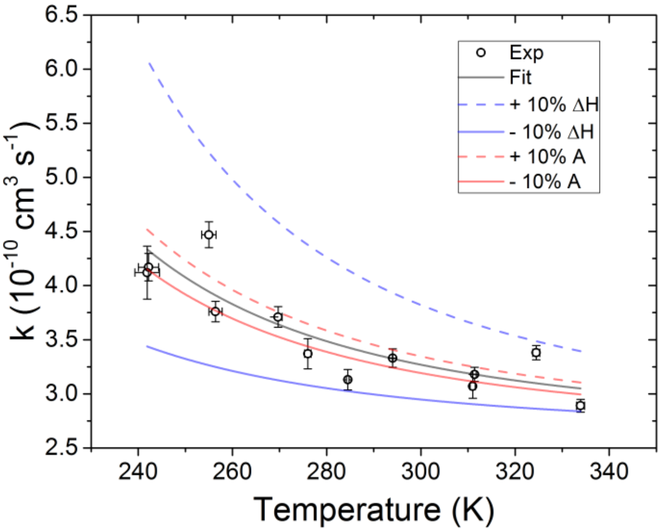

Figure S5: Sensitivity test for fit of equation (S6) to the temperature-dependent $CH_2OO + CF_3COOH$ reaction rate coefficients. The solid black line shows the optimized fit, and reproduces the fit shown in Figure 2 of the main article. The solid and dotted blue lines show k(T) values obtained using a fixed value of $A = 3.8 \times 10^{-18}$ cm^3 s^{-1} K^{-2} and +/- 10% changes in ΔH about the value reported in Table S3. The solid and dotted red lines show k(T) values obtained using a fixed value of ΔH = 13.5 kJ mol^{-1} and +/- 10% changes in A about the value listed in Table S3.

The fitted k_{3b} values of 2.5×10^{-10} cm^3 s^{-1} and 5.2×10^{-10} cm^3 s^{-1} correspond to C values of 1.0 and 2.3 at 294 K using Eq. (2) for the $CH_2OO + CF_3COOH$ and $(CH_3)_2COO + CF_3COOH$ reactions, respectively. Both of these C values show significant deviation from isotropic capture and suggest anisotropic capture in the non-adiabatic limit.[5] The fitted ΔH values obtained for $CH_2OO + CF_3COOH$ are in good agreement with the computational study of Long *et al*., as described in the main text. However, in the case of the $(CH_3)_2COO + CF_3COOH$ reaction, the fitted ΔH value is significantly smaller than the 49 kJ mol^{-1} predicted by Kumar *et al*. for the isomerization pathway. The highest estimate of k_{1b} from our observations is 6×10^{-10} cm^3 s^{-1} which gives a ΔH value of 28 kJ mol^{-1}. Thus, we postulate the presence of other high energy pathways, starting from the DHB complex or another pre-reactive complex, which compete with the single hydrogen bonded complex pathway.

S3.4 Representative pseudo-first-order plots

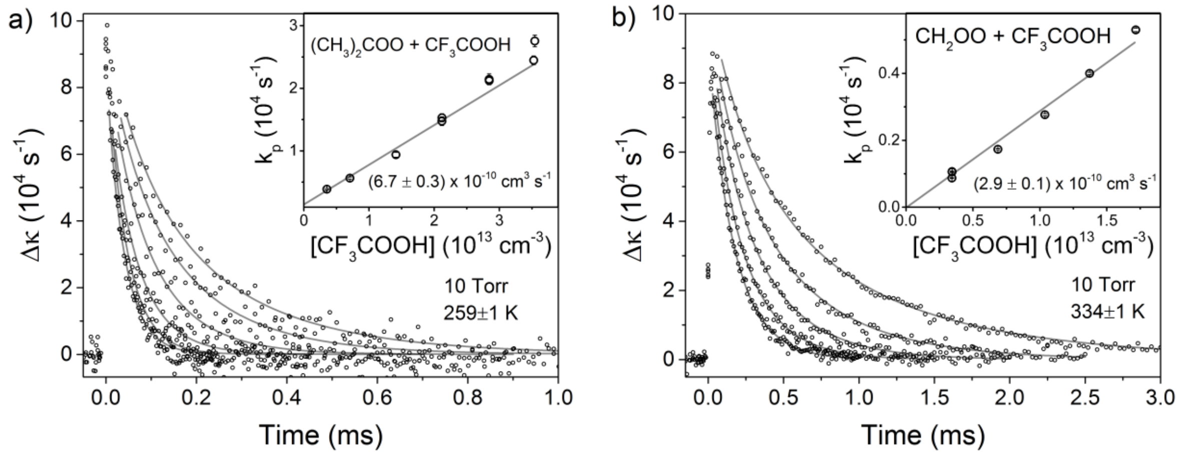

Figure S6: Representative pseudo-first-order fits for (a) $(CH_3)_2COO$ + CF_3COOH and (b) CH_2OO + CF_3COOH reaction rate coefficient measurements at different temperatures. Each decay trace corresponds to a different concentration of the TFA, as shown in the inset plots of pseudo-first-order rate coefficients against TFA concentration.

S4 Quantum chemistry calculations

Stationary point calculations of reactant, intermediates and product structures and energies were performed at the B3LYP/6-31+G(d) level of theory. Vibrational frequency calculations at the same level of theory verified whether these structures corresponded to maxima or minima along the reaction pathways. Intrinsic reaction coordinate (IRC) and relaxed dihedral angle calculations were performed to verify connectivity of the minima, and are reported in Section S4.3. All these calculations were performed using the Gaussian 09 program.[7]

Energy calculations were performed at the DF-HF//DF-LCCSD(T)-F12a/aug-cc-pVTZ level of theory using the B3LYP/6-31+G(d) geometries. Density fitting and local correlation approximations were used to reduce computational expense for these systems, while still providing a high degree of accuracy. Single point energy calculations were performed on each of the structures identified as stationary points after optimization using density functional theory. These energies were corrected for enthalpy and Gibbs free energy using values taken from DFT vibrational frequency calculations at standard temperature and pressure. All single point energy calculations were undertaken using the Molpro computational package.[8]

S4.1 Criegee intermediate structures

Figure S7 shows the structures of trifluoroacetic acid and the different Criegee intermediates studied. The two isoprene derived Criegee intermediates, anti-methacrolein oxide and syn-methyl vinyl ketone oxide, were chosen because they are formed in high yield by isoprene ozonolysis.

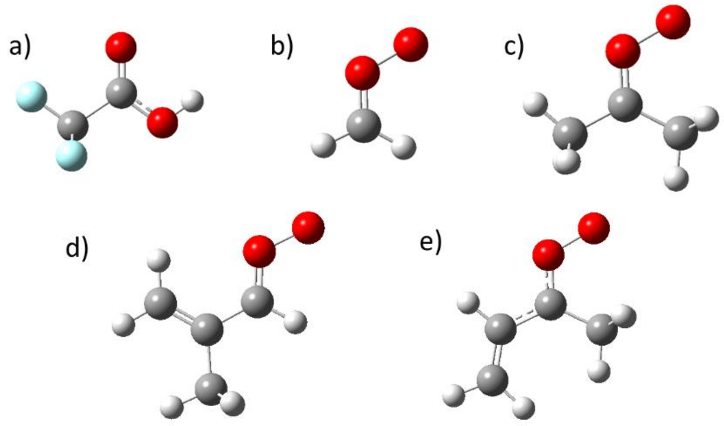

Figure S7: Optimized geometries of a) CF_3COOH, b) CH_2OO, c) $(CH_3)_2COO$, d) anti-C((trans-CH_3)=CH_2)-CHOO (anti-methacrolein oxide, anti-MACROO) and e) syn-CH_3-trans-(CH=CH_2)COO (syn-methyl vinyl ketone oxide, syn-MVKOO) calculated at the B3LYP/6-31+G(d) level of theory (white = H atom, grey = C atom, red = O atom and light blue = F atom).

S4.2 Reaction paths for isoprene derived Criegee intermediates with CF_3COOH

The computational methodology used in the studies of CF_3COOH reactions with CH_2OO and $(CH_3)_2COO$ can be applied to its reactions with larger Criegee intermediates expected to arise naturally from the ozonolysis of biogenic isoprene. This ozonolysis can occur through ozone addition at either the 1,2 or 3,4 locations, resulting in syn-methyl vinyl ketone oxide and anti-methacolein oxide, respectively. Computed pathways for reactions of these Criegee intermediates with CF_3COOH are shown in Figure S8, and do not exhibit any energetic barriers, but do reveal three submerged entropic barriers. These reactions proceed by formation of a planar complex, RCIIIa or RCIVa, analogous to those for the formaldehyde oxide and acetone oxide reactions, with binding free energies of ~25 kJ/mol. The Criegee moieties in these complexes then rotate over two small barriers until the Criegee carbonyl C atom is positioned close to the carbonyl O atom of CF_3COOH (complexes RCIIIc and RCIVc). Small entropic barriers of 1 and 4 kJ mol^{-1}, respectively, separate these complexes from adduct products containing new OH and OC bonds. The similarities in reaction profiles to those for CH_2OO and $(CH_3)_2OO$ reactions with CF_3COOH suggest that the isoprene-derived Criegee intermediate reactions will also be fast, and approach the dipole-capture limited value. The dipole moments for these larger Criegee intermediates are greater than for CH_2OO and $(CH_3)_2COO$, which should result in larger capture cross sections. The reaction path energy profiles suggest that the temperature dependences of these reactions will be similar to the $(CH_3)_2COO$ + CF_3COOH reaction and will lead directly to adduct formation without stabilization of complexes.

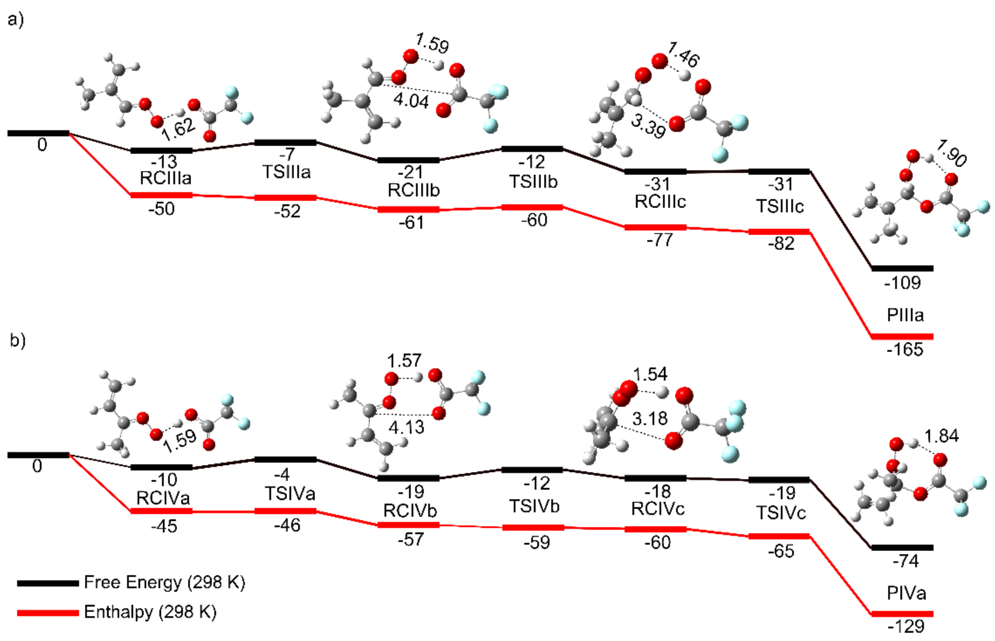

Figure S8: Computed pathways for the reactions of a) anti-methacrolein oxide and b) syn-methyl vinyl ketone oxide with CF_3COOH, calculated at the DF-HF//DF-LCCSD(T)-F12a/aug-cc-pVTZ//B3LYP-6-31+G(d) level of theory. Enthalpies and Gibbs free energies are specified relative to separated reactants (far left side). The pre-reaction complexes (RC), transition states (TS) and products (P) are discussed in the main text.

S4.2 Geometry and vibrational frequencies of different stationary points along the reaction pathways

Listed below are the geometries and vibrational frequencies of stationary points along the different reaction pathways computed at B3LYP/6-31+G(d) level of theory.

CH_2OO

Atomic No.	X	Y	z
8	-1.183863	-0.201247	0.000000
8	0.000000	0.460517	0.000000
6	1.075580	-0.197703	0.000000
1	1.034286	-1.284441	0.000000
1	1.983139	0.396497	0.000000

Vibrational frequencies (cm^{-1})

527.6 669.0 921.0 928.3 1248.3 1411.6 1552.5 3155.5 3305.0

Syn-CF_3COOH

6	-0.599520	-0.000512	-0.000004
6	0.942944	0.160285	-0.000002
8	1.501245	1.225567	-0.000049
8	1.527201	-1.045042	0.000031
1	2.495280	-0.915255	0.000041
9	-1.194053	1.195942	-0.000260
9	-1.002060	-0.680385	1.092984
9	-1.002042	-0.680844	-1.092709

Vibrational frequencies (cm^{-1})

25.6	236.5	241.1	384.5	416.3	496.9	576.5	605.2	658.9	779.8
787.3	1145.7	1167.3	1198.8	1253.8	1414.6	1865.5	3682.7		

RCIa

8	-2.703943	0.642165	0.098979
8	-3.489441	-0.477289	-0.030678
6	-4.733799	-0.319121	-0.058483
1	-5.146005	0.684921	0.020203
1	-5.313854	-1.230944	-0.162858
6	0.749478	0.517380	-0.004207
8	-0.225404	-0.372561	0.074641
1	-1.128600	0.077772	0.083493
8	0.654982	1.720189	-0.060761
6	2.126187	-0.201557	-0.008307
9	2.319376	-0.867662	1.153208
9	2.203052	-1.098671	-1.016861
9	3.127318	0.676112	-0.155493

Vibrational frequencies (cm^{-1})

17.9	25.8	34.7	51.1	80.2	100.7	169.0	256.3	278.0	395.4
428.6	507.6	550.3	585.3	677.4	696.7	770.2	802.9	888.3	956.0
998.1	1152.8	1174.5	1211.7	1247.7	1339.0	1425.5	1482.3	1582.4	1840.0
3061.2	3154.5	3304.4							

TSIa

8	-2.713589	0.552021	0.450653
8	-3.434311	-0.450415	-0.150091
6	-4.673256	-0.291097	-0.268425
1	-5.131777	0.623091	0.103266
1	-5.200740	-1.108825	-0.749851
6	0.722815	0.503002	0.015715
8	-0.203649	-0.377281	0.355700
1	-1.119842	0.045321	0.381132
8	0.579042	1.675365	-0.236759
6	2.114534	-0.184798	-0.033961
9	2.420745	-0.748110	1.155699
9	2.130055	-1.161497	-0.970500
9	3.076741	0.696301	-0.337481

Vibrational frequencies (cm^{-1})

-21.6	27.7	32.9	49.9	85.9	100.5	167.9	258.0	276.3	395.0
428.3	508.1	549.2	584.7	676.9	691.4	772.1	800.0	890.7	995.9
998.0	1153.2	1174.5	1210.8	1248.3	1327.5	1425.8	1466.7	1582.0	1839.5
3060.9	3154.7	3304.6							

PIa

8	3.051523	-0.922783	-0.064846
8	2.651247	0.323293	0.564551
6	1.896825	1.073511	-0.309326
1	2.109637	0.816307	-1.348681
1	2.074477	2.124416	-0.081184
6	-0.164411	-0.215965	-0.105434
8	0.334887	-1.315281	-0.200623
1	2.213666	-1.437357	-0.006903
8	0.436106	0.963047	-0.100031
6	-1.699262	-0.023272	0.037723
9	-2.178295	0.688665	-1.003748
9	-2.319795	-1.206295	0.064230
9	-1.989332	0.640385	1.173582

Vibrational frequencies (cm^{-1})

24.7	60.7	110.0	146.9	212.2	234.2	283.2	340.6	425.2	460.1
507.6	547.1	556.8	582.6	734.2	774.6	816.2	863.6	912.8	1091.9
1147.4	1166.0	1170.8	1228.0	1311.7	1354.3	1441.0	1482.2	1483.0	1818.9
3115.2	3197.0	3541.1							

$(CH_3)_2COO$

6	1.758476	-0.544819	-0.000085
1	2.313700	-0.197747	-0.881805
1	1.726296	-1.637045	-0.001072
1	2.313121	-0.199622	0.882839
6	0.380577	0.026656	0.000096
6	0.071382	1.473646	0.000073
1	0.980555	2.079015	-0.000047
1	-0.556328	1.708877	-0.870423
1	-0.556807	1.708481	0.870258
8	-0.570089	-0.818997	0.000050
8	-1.865304	-0.330360	-0.000081

Vibrational frequencies (cm^{-1})

154.0	180.2	275.1	304.9	360.2	475.5	597.1	815.3	929.5	944.5
991.2	1078.7	1102.4	1318.6	1415.1	1429.9	1463.0	1477.2	1489.3	1496.8
1570.1	3038.1	3043.2	3085.8	3087.6	3155.9	3157.0			

RCIIa

8	-1.720337	0.728046	0.079462
8	-2.546289	-0.402004	0.016990
6	-3.796332	-0.182285	-0.010042
6	1.653278	0.514926	-0.022750
8	0.661247	-0.344982	0.083528
1	-0.249412	0.125872	0.078230
8	1.595471	1.719612	-0.118488
6	3.014360	-0.234872	-0.003767
9	3.076744	-1.158591	-0.990422

9	4.038948	0.613965	-0.167945
9	3.191372	-0.878665	1.173902
6	-4.658509	-1.394868	-0.077173
1	-5.335637	-1.415974	0.786719
1	-5.287887	-1.352258	-0.975686
1	-4.057656	-2.306643	-0.093882
6	-4.305726	1.205042	0.024930
1	-5.396469	1.232249	-0.004549
1	-3.924199	1.705848	0.924880
1	-3.875480	1.767490	-0.814653

Vibrational frequencies (cm^{-1})

9.3	22.3	25.0	28.5	70.7	94.6	137.0	153.4	187.2	257.3
264.3	279.4	355.5	368.9	401.5	441.3	489.0	508.2	581.3	611.9
701.2	773.4	805.1	819.9	915.8	932.8	994.8	1027.0	1083.1	1108.4
1149.4	1178.4	1210.4	1319.7	1362.2	1409.6	1428.9	1465.1	1476.5	1487.1
1496.7	1512.5	1590.5	1828.9	2775.3	3046.0	3050.4	3095.6	3100.0	3166.5
3168.9									

TSIIa

8	1.729606	-0.735289	0.340619
8	2.515945	0.391599	0.070013
6	3.764536	0.191092	-0.042261
6	-1.625466	-0.497473	-0.096074
8	-0.677095	0.278404	0.387144
1	0.243820	-0.169839	0.343720
8	-1.524616	-1.612946	-0.553419
6	-2.999711	0.223078	-0.007451
9	-2.980735	1.391309	-0.691039
9	-3.980370	-0.536333	-0.516433
9	-3.314817	0.504046	1.278040
6	4.583614	1.400367	-0.333546
1	5.334535	1.538505	0.455299
1	5.130840	1.258330	-1.274641
1	3.958751	2.293182	-0.404786
6	4.312897	-1.172772	0.114256
1	5.396890	-1.185740	-0.012634
1	4.028535	-1.563919	1.100365
1	3.823980	-1.841623	-0.606839

Vibrational frequencies (cm^{-1})

-9.1	22.6	25.3	27.4	74.9	95.2	135.9	155.0	191.0	258.1
264.9	279.4	355.3	368.9	400.8	439.8	489.8	508.3	581.3	611.8
697.8	774.2	803.1	818.0	917.6	933.7	995.3	1059.5	1086.4	1108.6
1149.9	1178.5	1209.9	1320.2	1354.9	1410.4	1429.3	1465.7	1476.4	1487.6
1493.5	1497.4	1590.0	1828.9	2776.4	3046.0	3050.5	3095.7	3099.9	3166.6
3169.0									

RCIIb

8	1.826777	1.791234	-0.076248
8	2.299556	0.647301	-0.764989
6	2.794992	-0.274690	-0.049725
6	-0.931025	0.097271	0.065446
8	-0.693574	1.345338	-0.238685
1	0.329370	1.558955	-0.193543
8	-0.128898	-0.773038	0.356146
6	-2.453303	-0.208251	0.008934
9	-2.927076	-0.027041	-1.243821
9	-2.706880	-1.475958	0.368560
9	-3.141316	0.606572	0.837065
6	3.235861	-1.485144	-0.794431
1	4.293194	-1.689837	-0.585489
1	2.657954	-2.345547	-0.432504
1	3.081394	-1.369393	-1.869009
6	2.920649	-0.124625	1.415720
1	3.476418	-0.957622	1.850558
1	3.393852	0.837250	1.645293
1	1.911332	-0.080010	1.843014

Vibrational frequencies (cm^{-1})

16.2	40.2	48.8	73.0	96.4	108.9	138.5	153.2	185.0	261.2
280.3	311.9	349.8	371.6	402.3	433.8	477.4	509.1	581.4	590.6
702.8	782.0	810.3	813.2	903.1	924.0	995.0	1081.6	1110.8	1124.0
1161.8	1187.9	1210.6	1318.0	1383.6	1400.7	1422.3	1468.7	1476.1	1487.3
1497.9	1519.9	1598.1	1783.7	2405.4	3051.8	3061.7	3108.3	3119.6	3168.5
3170.2									

TSIIb

8	1.854616	1.873521	-0.104170
8	2.264030	0.674442	-0.778011
6	2.472733	-0.334134	-0.028364
6	-0.775691	0.139646	0.040880
8	-0.533201	1.387093	-0.129644
1	0.632073	1.670026	-0.125718
8	0.021210	-0.790039	0.202972
6	-2.297975	-0.179206	0.008363
9	-2.789152	0.022011	-1.236409
9	-2.549432	-1.455889	0.346776
9	-2.982158	0.615014	0.858531
6	2.815973	-1.581878	-0.763549
1	3.793388	-1.950976	-0.429129
1	2.064668	-2.338287	-0.510113
1	2.822072	-1.419320	-1.842971
6	2.583272	-0.235461	1.447655
1	2.459350	-1.220370	1.901000
1	3.597027	0.134758	1.668309
1	1.874979	0.480007	1.859464

Vibrational frequencies (cm^{-1})

-572.4	13.3	49.8	71.1	93.5	114.2	144.0	157.3	165.1	228.7
269.6	301.9	333.9	377.4	386.4	425.5	476.3	510.9	561.8	576.3
665.2	726.6	791.6	805.9	836.4	919.2	937.3	983.9	1084.4	1109.1
1153.6	1193.5	1209.7	1224.0	1322.1	1416.4	1420.0	1448.6	1465.9	1475.8
1482.3	1500.8	1586.5	1661.4	1773.8	3037.0	3061.0	3125.8	3147.8	3174.2
3214.5									

PIIa

8	-2.308424	-1.784738	-0.151317
8	-2.161226	-0.509475	-0.824218
6	-1.718072	0.502247	0.019997
6	0.626908	-0.415781	-0.015008
8	0.382600	-1.605531	-0.028862
1	-1.373248	-2.098585	-0.172757
8	-0.191282	0.612269	-0.002714
6	2.096939	0.094031	-0.002534
9	2.954751	-0.928548	-0.080987
9	2.327254	0.924115	-1.039889
9	2.345673	0.770225	1.140680
6	-2.145692	1.800827	-0.649003
1	-3.232309	1.899415	-0.576957
1	-1.668484	2.650774	-0.154986
1	-1.858272	1.793561	-1.704086
6	-2.124543	0.372503	1.477550
1	-1.802112	1.264693	2.021128
1	-3.213524	0.286624	1.536735
1	-1.687747	-0.511775	1.943570

Vibrational frequencies (cm^{-1})

18.3	55.9	65.3	131.2	209.3	220.4	225.1	242.5	256.9	295.9
302.8	346.3	371.2	434.5	444.3	504.6	520.5	555.3	567.7	620.4
684.0	738.7	774.7	807.2	871.9	944.2	957.3	994.6	1038.3	1123.7
1161.1	1191.6	1227.0	1258.3	1294.1	1388.0	1430.4	1444.0	1487.2	1499.1
1500.2	1514.3	1521.2	1800.9	3077.4	3083.4	3152.3	3156.2	3162.1	3174.0
3505.2									

anti-trans-C(CH_3)=CH_2)-CHOO

6	2.074602	-0.940310	-0.000082
1	2.015553	-1.588109	0.883741
1	2.014895	-1.588708	-0.883376
1	3.055767	-0.457702	-0.000584
6	0.968197	0.091895	0.000052
6	-0.379275	-0.448006	0.000113
1	-0.585473	-1.518450	0.000481
6	1.201150	1.422207	0.000095
1	0.391345	2.145188	0.000265
1	2.215829	1.809118	-0.000309

8	-1.396908	0.315427	-0.000179
8	-2.640087	-0.259933	0.000018

Vibrational frequencies (cm^{-1})

95.2	144.0	196.9	198.5	368.7	456.1	493.5	556.2	673.6	900.0
957.7	961.8	977.7	1021.7	1050.9	1084.6	1279.5	1375.8	1439.2	1461.6
1505.6	1515.7	1528.6	1673.8	3044.8	3095.7	3138.7	3179.3	3181.6	3266.6

RCIIIa

6	3.330497	-0.604324	0.025875
6	4.383803	0.392473	-0.020782
6	4.089554	1.710055	-0.068995
1	3.065175	2.070061	-0.074177
1	4.878795	2.455095	-0.104837
8	2.106859	-0.273614	0.034827
8	1.182764	-1.314463	0.075504
1	-0.230556	-0.531215	0.079373
8	-2.312051	-1.793589	-0.151787
6	-2.169366	-0.599133	-0.031853
8	-1.046375	0.081213	0.092493
6	-3.383995	0.369453	-0.000323
9	-4.537425	-0.293401	-0.161565
9	-3.442408	1.028298	1.180475
9	-3.293395	1.294996	-0.983327
1	3.535830	-1.675024	0.054909
6	5.794948	-0.151905	-0.012668
1	5.973022	-0.811074	-0.871274
1	6.522884	0.662380	-0.055330
1	5.991677	-0.735359	0.895268

Vibrational frequencies (cm^{-1})

11.4	22.8	26.3	29.4	64.3	86.8	109.7	128.0	143.3	198.2
225.3	257.5	288.6	371.8	395.5	426.6	486.2	487.4	507.9	561.0
585.0	666.5	699.5	772.4	805.7	893.7	948.9	990.3	999.4	1006.7
1025.8	1047.9	1087.7	1149.6	1176.9	1211.2	1282.4	1353.0	1393.4	1441.8
1464.1	1501.8	1506.2	1517.4	1548.4	1676.8	1833.6	2858.6	3049.5	3102.6
3144.7	3181.8	3183.5	3270.2						

TSIIIa

6	3.270623	-0.605450	0.126187
6	4.251476	0.437130	-0.110380
6	3.885908	1.736986	-0.150025
1	2.855635	2.048876	-0.007807
1	4.621352	2.516096	-0.327159
8	2.041988	-0.334607	0.281078
8	1.192307	-1.412834	0.510192
1	-0.258948	-0.706933	0.548342
8	-2.044102	-1.487495	-0.944775

6	-2.064562	-0.561482	-0.167746
8	-1.108756	-0.149851	0.642353
6	-3.312759	0.353066	-0.023969
9	-4.344269	-0.118522	-0.738003
9	-3.707621	0.455302	1.264019
9	-3.033863	1.602470	-0.469955
1	3.537556	-1.661359	0.181913
6	5.674246	-0.039775	-0.300713
1	5.757845	-0.709824	-1.165270
1	6.346379	0.806747	-0.463369
1	6.030878	-0.591393	0.577909

Vibrational frequencies (cm^{-1})

-17.8	13.8	21.1	23.3	72.2	86.5	109.0	127.9	143.8	197.2
226.6	258.7	284.0	371.2	395.5	426.7	481.3	486.6	508.0	560.9
584.0	665.3	693.1	773.6	800.5	894.3	950.2	988.8	1001.4	1024.6
1048.1	1075.7	1087.6	1148.7	1178.2	1210.4	1282.0	1334.5	1393.8	1442.3
1463.3	1469.0	1506.4	1516.9	1548.2	1676.2	1832.4	2851.4	3049.5	3102.5
3144.7	3181.6	3183.8	3270.0						

RCIIIb

6	3.024809	0.733716	-0.074353
6	3.702425	-0.545228	0.022539
6	2.978751	-1.684793	0.097378
1	1.891803	-1.675195	0.084081
1	3.473087	-2.650204	0.158235
8	1.778753	0.811032	0.130773
8	1.172716	2.052570	-0.047811
1	-0.346269	1.592547	0.026338
8	-0.699069	-0.828594	-0.266045
6	-1.526920	0.039960	-0.083549
8	-1.339228	1.329461	0.075163
6	-3.040851	-0.299685	0.008936
9	-3.261427	-1.597631	-0.249995
9	-3.509704	-0.037097	1.250579
9	-3.761951	0.429334	-0.869701
1	3.536630	1.663465	-0.327139
6	5.213778	-0.499742	-0.006351
1	5.614345	0.059227	0.848372
1	5.629816	-1.510125	0.026143
1	5.581013	-0.012281	-0.918227

Vibrational frequencies (cm^{-1})

15.4	21.9	30.7	42.0	71.0	99.9	106.3	136.2	140.9	199.7
247.0	260.9	279.9	368.7	400.0	430.0	478.6	487.6	508.7	557.6
584.3	680.3	702.6	777.3	811.2	895.3	955.1	999.0	1016.4	1024.7
1041.5	1048.3	1087.5	1156.1	1183.9	1209.0	1281.8	1367.0	1396.5	1440.6
1466.1	1506.0	1516.2	1522.3	1555.0	1672.3	1811.2	2709.7	3047.8	3100.0
3142.4	3172.5	3178.2	3264.5						

TSIIIb

6	2.736663	0.633715	-0.256797
6	3.489090	-0.559892	0.079498
6	3.035934	-1.400992	1.033465
1	2.107058	-1.215829	1.563395
1	3.579485	-2.308490	1.279943
8	1.814686	1.063707	0.493940
8	1.128050	2.199103	0.037786
1	-0.364934	1.710793	0.158370
8	-0.510981	-0.652363	-0.410763
6	-1.414314	0.104367	-0.117329
8	-1.338383	1.376440	0.196732
6	-2.889106	-0.381797	-0.063864
9	-2.983320	-1.678156	-0.396626
9	-3.396450	-0.233256	1.180734
9	-3.663495	0.325432	-0.915265
1	2.929350	1.214487	-1.159958
6	4.740687	-0.800583	-0.734633
1	5.472247	0.004590	-0.593937
1	5.213720	-1.742393	-0.444618
1	4.511763	-0.853336	-1.806382

Vibrational frequencies (cm^{-1})

-35.2	16.7	24.3	37.8	66.5	98.8	114.1	133.3	143.5	212.7
247.6	262.9	284.2	365.3	398.6	427.3	469.1	481.4	508.4	555.6
583.8	661.4	699.6	777.8	808.6	885.6	937.8	993.4	1007.5	1024.7
1045.2	1064.7	1087.9	1157.1	1183.7	1209.1	1282.6	1366.7	1396.8	1440.2
1463.3	1504.9	1508.7	1516.7	1559.7	1679.3	1807.9	2669.4	3048.5	3101.5
3143.4	3179.8	3183.0	3273.9						

RCIIIc

6	2.203785	0.411122	-0.457567
6	3.012881	-0.666414	0.090459
6	3.688226	-0.491637	1.244517
1	3.648839	0.442744	1.796109
1	4.302748	-1.287268	1.655727
8	1.964219	1.458973	0.212605
8	1.144989	2.413437	-0.450691
1	-0.191403	1.894087	-0.171424
8	-0.218082	-0.562581	-0.340245
6	-1.160204	0.183138	-0.110139
8	-1.157876	1.477893	-0.000918
6	-2.584351	-0.399614	0.105202
9	-2.596808	-1.729796	-0.070017
9	-3.016432	-0.139434	1.358436
9	-3.462384	0.144720	-0.762534
1	1.810406	0.377497	-1.470325
6	3.030807	-1.938504	-0.723155

1	3.410491	-1.757887	-1.736780
1	3.668195	-2.690749	-0.250833
1	2.018481	-2.348156	-0.817344

Vibrational frequencies (cm^{-1})

11.3	30.7	41.3	60.0	74.7	109.1	138.3	160.7	202.2	233.4
256.7	284.5	318.3	365.4	398.4	430.3	450.1	497.7	510.8	552.1
583.8	673.9	708.1	783.6	820.8	877.4	927.1	991.4	997.7	1027.3
1045.2	1089.8	1134.2	1165.7	1191.2	1212.2	1284.8	1390.0	1403.9	1439.1
1462.8	1502.9	1519.3	1542.5	1557.9	1686.6	1725.8	2084.9	3050.7	3109.2
3143.7	3180.3	3223.2	3268.3						

TSIIIc

6	2.125811	0.418330	-0.449452
6	2.903838	-0.689855	0.085045
6	3.573850	-0.547012	1.246012
1	3.547785	0.377865	1.814103
1	4.171666	-1.360609	1.646597
8	1.928234	1.469505	0.234858
8	1.123736	2.446940	-0.433952
1	-0.090530	1.952926	-0.196585
8	-0.151541	-0.550047	-0.331545
6	-1.099305	0.204502	-0.113546
8	-1.102563	1.489244	-0.019851
6	-2.515432	-0.399175	0.104238
9	-3.409235	0.151634	-0.742716
9	-2.521422	-1.727285	-0.093471
9	-2.935161	-0.165211	1.367454
1	1.766131	0.424411	-1.474410
6	2.899427	-1.947221	-0.750071
1	3.282330	-1.756242	-1.760583
1	3.523611	-2.718203	-0.290469
1	1.879311	-2.334946	-0.849483

Vibrational frequencies (cm^{-1})

-186.6	11.7	34.4	62.3	74.9	88.2	125.0	159.4	203.4	231.6
262.1	298.8	312.1	361.0	369.3	426.9	450.8	507.7	512.0	551.7
579.5	678.3	712.2	787.8	830.9	866.2	913.0	988.9	997.2	1028.6
1043.7	1090.3	1117.7	1157.9	1200.3	1209.2	1213.2	1284.9	1395.5	1435.5
1438.8	1463.0	1502.5	1519.9	1554.2	1611.8	1688.2	1792.7	3051.5	3111.7
3144.5	3180.5	3230.2	3268.5						

PIIIa

6	1.458702	0.242546	-0.302771
6	2.393651	-0.907891	-0.003991
6	3.482160	-0.712693	0.747893

1	3.709256	0.249850	1.192292
1	4.178226	-1.527008	0.931008
8	1.724414	1.347950	0.485886
8	1.581856	2.564345	-0.293191
1	0.601043	2.653611	-0.309268
8	0.083562	-0.295426	-0.021714
6	-0.972032	0.487651	-0.142084
8	-1.013431	1.666786	-0.420829
6	-2.266159	-0.330657	0.125119
9	-2.403120	-1.297523	-0.808622
9	-2.219831	-0.918543	1.336171
9	-3.343233	0.459095	0.076033
1	1.456133	0.528734	-1.358801
6	2.048234	-2.223270	-0.656891
1	1.906891	-2.106677	-1.740289
1	2.848789	-2.951069	-0.496074
1	1.116771	-2.638051	-0.255956

Vibrational frequencies (cm^{-1})

25.9	46.3	59.8	87.1	99.5	176.0	189.0	221.7	230.6	248.1
282.3	336.6	369.3	424.0	451.4	479.5	504.0	520.5	552.3	570.1
622.5	716.3	735.8	776.2	831.0	853.8	920.8	955.0	969.8	1009.0
1031.8	1090.5	1114.2	1162.1	1175.9	1225.5	1274.9	1318.4	1384.6	1395.4
1437.5	1462.2	1475.6	1503.7	1517.9	1725.2	1810.5	3040.7	3099.9	3102.8
3136.1	3177.6	3268.0	3541.0						

Syn-CH_3-trans-($CH=CH_2$)COO

6	-0.286528	1.458523	-0.000019
1	0.632589	2.044972	-0.001047
1	-0.902452	1.710721	0.872783
1	-0.904272	1.710279	-0.871645
6	-0.022469	0.001434	-0.000014
6	1.262054	-0.664207	-0.000065
1	1.209510	-1.750996	-0.000085
6	2.457818	-0.044775	0.000013
1	2.562114	1.036053	0.000083
1	3.377629	-0.621119	0.000060
8	-1.022948	-0.809284	0.000088
8	-2.282099	-0.270186	-0.000042

Vibrational frequencies (cm^{-1})

119.6	187.2	256.8	277.9	330.8	457.2	492.3	600.9	678.9	809.1
957.4	971.0	1019.0	1027.5	1048.8	1073.6	1315.4	1346.1	1418.1	1466.9
1470.9	1496.8	1507.0	1677.0	3053.3	3097.7	3175.1	3183.3	3191.2	3265.6

RCIVa

6	3.974318	-1.668224	0.028336
1	5.061813	-1.756893	0.014095

1	3.529194	-2.195313	-0.825898
1	3.555015	-2.159993	0.916058
6	3.543473	-0.252185	0.006008
6	4.464136	0.874467	-0.030791
1	5.516781	0.605394	-0.037760
6	4.091801	2.167784	-0.056061
1	3.047876	2.467912	-0.050493
1	4.837455	2.956270	-0.083294
8	2.295158	0.026205	0.018803
8	1.420225	-1.061608	0.051714
1	-0.009633	-0.359428	0.060190
8	-2.001742	-1.766429	-0.144516
6	-1.942370	-0.563397	-0.033395
8	-0.870907	0.195418	0.075964
6	-3.223913	0.314921	0.003833
9	-3.209230	1.240158	-0.983667
9	-4.328048	-0.430108	-0.144988
9	-3.321058	0.973636	1.182412

Vibrational frequencies (cm^{-1})

12.1	21.9	24.4	29.5	70.5	76.5	88.3	131.5	193.7	240.0
256.7	266.5	276.9	360.5	400.8	437.5	453.2	494.6	508.2	583.0
618.6	666.0	700.9	773.3	803.9	815.1	937.5	1008.3	1014.4	1027.0
1041.5	1049.1	1111.5	1149.2	1177.9	1211.2	1281.2	1346.9	1361.3	1414.4
1457.8	1465.9	1500.7	1510.2	1528.4	1681.9	1829.8	2779.5	3050.6	3096.9
3170.2	3182.8	3205.2	3272.9						

TSIVa

6	3.976401	-1.612802	-0.009894
1	5.043798	-1.653407	-0.232850
1	3.402252	-2.203082	-0.736317
1	3.765764	-2.074938	0.963612
6	3.473425	-0.220658	-0.008748
6	4.309971	0.943832	-0.258612
1	5.350942	0.721592	-0.476626
6	3.876796	2.218009	-0.230259
1	2.843365	2.470817	-0.011464
1	4.560165	3.038033	-0.427741
8	2.234979	0.000942	0.222154
8	1.442357	-1.120778	0.470358
1	-0.022853	-0.502318	0.529946
8	-1.765635	-1.470003	-0.904112
6	-1.849317	-0.526229	-0.152078
8	-0.917835	-0.011893	0.624825
6	-3.170552	0.278956	-0.004805
9	-3.008159	1.543607	-0.465030
9	-4.164570	-0.288258	-0.702724
9	-3.560016	0.359152	1.286867

Vibrational frequencies (cm^{-1})

-18.5	13.7	21.2	20.7	76.8	78.0	91.4	130.6	190.5	239.8
258.4	265.7	274.0	358.8	399.7	435.1	451.7	493.0	508.0	582.3
618.8	665.2	695.0	774.3	801.3	811.2	938.7	1007.7	1013.6	1038.7
1048.8	1094.5	1113.9	1149.2	1178.7	1210.3	1281.8	1345.2	1346.4	1413.1
1456.6	1464.6	1476.2	1502.8	1524.8	1681.9	1828.7	2772.2	3050.0	3096.5
3169.9	3183.2	3204.6	3273.3						

RCIVb

6	4.154841	-1.291481	0.001251
1	5.199644	-0.976569	0.001729
1	3.940751	-1.922495	-0.871139
1	3.939669	-1.921427	0.874151
6	3.235991	-0.129742	-0.000010
6	3.667353	1.260062	-0.000280
1	4.743073	1.413092	-0.000281
6	2.817927	2.306089	-0.000359
1	1.738769	2.170412	-0.000210
1	3.199336	3.322751	-0.000531
8	1.973819	-0.319275	-0.000887
8	1.523772	-1.641166	-0.000305
1	-0.022327	-1.368886	-0.000605
8	-0.732211	1.002267	0.000599
6	-1.423009	0.004043	-0.000051
8	-1.048518	-1.253016	-0.000734
6	-2.974584	0.102465	0.000061
9	-3.496368	-0.498440	1.093065
9	-3.378578	1.382099	-0.000070
9	-3.496711	-0.498766	-1.092569

Vibrational frequencies (cm^{-1})

15.2	18.6	31.5	36.8	75.0	101.4	103.8	138.8	183.2	258.4
260.1	268.5	276.0	355.2	400.6	433.9	455.5	493.6	507.8	583.6
618.7	667.9	703.5	777.5	805.0	820.0	943.1	1008.5	1038.8	1041.9
1049.1	1052.8	1117.8	1155.4	1183.5	1209.2	1283.2	1348.2	1373.0	1416.5
1458.3	1468.9	1502.4	1521.0	1539.0	1676.9	1808.4	2634.4	3052.31	3100.5
3170.2	3173.0	3204.2	3267.5						

TSIVb

6	3.410725	-1.166205	1.086611
1	4.207668	-0.782989	1.725989
1	3.736450	-2.064189	0.546947
1	2.551574	-1.485049	1.691304
6	2.951968	-0.142749	0.119980
6	3.474895	1.214720	0.075661

1	4.397816	1.376221	0.626271
6	2.854938	2.235762	-0.543010
1	1.904446	2.103953	-1.050931
1	3.282185	3.233795	-0.528171
8	2.044451	-0.430965	-0.730023
8	1.500251	-1.722611	-0.643506
1	-0.019605	-1.391154	-0.690054
8	-0.470112	0.600091	0.643170
6	-1.267920	-0.107313	0.061074
8	-1.030211	-1.172042	-0.666930
6	-2.792577	0.189118	0.111841
9	-3.461533	-0.835068	0.686845
9	-3.046593	1.299761	0.820764
9	-3.292733	0.365711	-1.131608

Vibrational frequencies (cm^{-1})

-30.5	16.4	27.1	36.0	72.0	96.2	109.6	151.6	183.6	244.0
261.3	275.1	289.0	354.0	400.0	430.0	447.1	496.5	508.3	582.9
610.6	662.2	700.0	778.4	806.1	810.0	927.1	1004.0	1013.0	1035.5
1049.3	1091.7	1109.0	1156.9	1183.9	1208.8	1280.9	1344.7	1370.8	1411.1
1458.3	1466.1	1500.6	1509.3	1536.4	1684.0	1804.6	2592.4	3054.4	3104.1
3170.3	3185.1	3202.2	3277.0						

RCIVc

6	2.615987	-1.037570	1.476813
1	3.199956	-0.627989	2.302846
1	2.963395	-2.042252	1.208043
1	1.564184	-1.146208	1.770018
6	2.677300	-0.159616	0.287639
6	3.365041	1.125455	0.278935
1	3.779134	1.430907	1.235518
6	3.501164	1.908060	-0.806288
1	3.099480	1.623680	-1.774411
1	4.023184	2.857742	-0.741455
8	2.122969	-0.513429	-0.807448
8	1.471925	-1.762637	-0.803867
1	-0.001984	-1.328907	-0.893903
8	-0.418430	0.306220	0.857168
6	-1.214253	-0.150944	0.058831
8	-0.994302	-1.021872	-0.894034
6	-2.705047	0.286309	0.071142
9	-3.517949	-0.776903	0.253691
9	-2.944289	1.165690	1.055617
9	-3.040627	0.870836	-1.100822

Vibrational frequencies (cm^{-1})

17.8	24.8	45.2	58.0	71.0	92.0	108.8	162.9	173.9	233.5
259.0	275.9	320.1	347.5	401.0	432.1	444.2	485.8	509.0	583.4

603.7	662.8	699.4	780.1	805.2	808.4	925.3	1009.3	1014.3	1040.4
1048.6	1107.6	1119.7	1159.4	1185.6	1210.7	1279.4	1347.0	1370.1	1404.5
1459.1	1468.6	1499.5	1504.1	1531.9	1686.3	1796.7	2513.8	3058.9	3114.2
3169.4	3183.5	3209.0	3273.4						

TSIVc

6	2.212516	-0.737048	1.664563
1	1.999996	0.001639	2.439202
1	3.199875	-1.183442	1.864734
1	1.473580	-1.534135	1.673606
6	2.294573	-0.075022	0.337979
6	2.831352	1.276376	0.204315
1	3.004508	1.795696	1.142029
6	3.096736	1.868274	-0.971717
1	2.918924	1.371253	-1.920725
1	3.497693	2.876738	-1.003968
8	2.094987	-0.698884	-0.765535
8	1.501685	-1.997305	-0.646995
1	0.344448	-1.663592	-0.683068
8	-0.145279	0.516787	0.588914
6	-0.983102	-0.172581	-0.002814
8	-0.815705	-1.255147	-0.665263
6	-2.472259	0.280587	0.025698
9	-2.635092	1.440285	0.686383
9	-2.948004	0.452295	-1.228028
9	-3.239506	-0.651610	0.636208

Vibrational frequencies (cm^{-1})

-570.9	11.7	33.0	54.9	67.9	97.3	111.1	129.3	167.7	210.6
240.9	268.6	293.8	354.0	388.1	422.1	444.8	478.5	511.1	555.0
596.0	644.2	675.9	724.0	790.8	805.1	835.5	932.9	1005.9	1012.7
1026.2	1051.9	1106.3	1150.7	1189.7	1209.4	1220.0	1290.2	1348.0	1419.9
1445.5	1448.6	1476.9	1496.8	1533.7	1664.9	1691.0	1772.0	3031.6	3148.3
3184.9	3214.0	3221.1	3274.7						

PIVa

6	1.787856	-0.376911	1.694803
1	1.552066	0.416149	2.409466
1	2.844821	-0.640739	1.787212
1	1.186308	-1.259124	1.920672
6	1.533758	0.135119	0.284174
6	2.159645	1.481511	0.008407
1	1.654227	2.314598	0.490977
6	3.255097	1.661752	-0.729926
1	3.760546	0.835650	-1.220201
1	3.671063	2.656141	-0.865202
8	1.903812	-0.713599	-0.747935
8	1.873912	-2.110767	-0.365329
1	0.911738	-2.296684	-0.480784

8	0.036750	0.471962	0.207501
6	-0.892006	-0.417588	-0.063359
8	-0.784928	-1.604114	-0.300587
6	-2.287402	0.270502	-0.052521
9	-2.343269	1.244402	-0.983261
9	-3.255260	-0.615921	-0.308740
9	-2.530228	0.827058	1.154133

Vibrational frequencies (cm^{-1})

20.9	55.0	60.4	107.6	120.1	197.6	220.9	225.7	249.8	262.6
282.3	334.4	339.1	374.6	433.2	472.8	511.0	515.9	554.6	593.8
614.4	678.9	706.9	736.7	774.1	801.7	868.9	933.0	977.6	984.6
1019.9	1062.6	1089.3	1162.3	1172.6	1216.0	1228.5	1274.6	1332.4	1384.8
1432.9	1459.6	1484.6	1504.1	1505.6	1717.3	1799.0	3082.9	3157.7	3169.8
3176.6	3197.9	3264.4	3505.1						

S4.3 Intrinsic Reaction Coordinate profiles

Figure S9, S10 and S11 shows IRC profiles connecting stationary points along the pathways of reactions of TFA with selected Criegee intermediates. For the rotational barriers, relaxed scans were performed along the dihedral angle responsible for the imaginary vibrational frequency mode. The starting and the final geometries of the dihedral and IRC scans were verified to optimize to the correct minima. In the CH_2OO + CF_3COOH reaction, the hydrogen atom transfer step is spontaneous. For the $(CH_3)_2COO$ + CF_3COOH reaction, RCIIb proceeds to PIIa via a low-lying TS (TSIIb) for H-atom transfer. Analogous behaviour is observed with TSIIIc and TSIVc for reactions of TFA with anti-trans-C(CH_3)=CH_2)-CHOO and syn-CH_3-trans-(CH=CH_2)COO respectively.

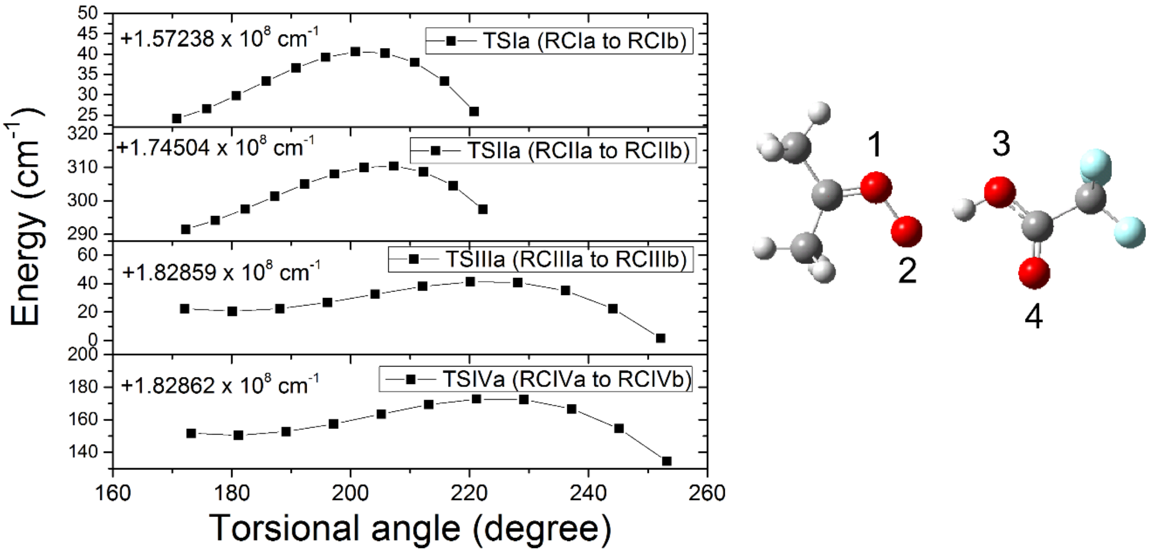

Figure S9: Relaxed scans calculated at the B3LYP/6-31+G(d) level of theory for reactions I, II, III and IV of CF_3COOH with CH_2OO, $(CH_3)_2COO$, anti-trans-C(CH_3)=CH_2)-CHOO and Syn-CH_3-trans-(CH=CH_2)COO respectively. The dihedral angle used

for the scan is shown by the labels 1, 2, 3 and 4 for the $(CH_3)_2COO$ + CF_3COOH reaction. The energies were shifted by the amounts labelled in the panels for clarity.

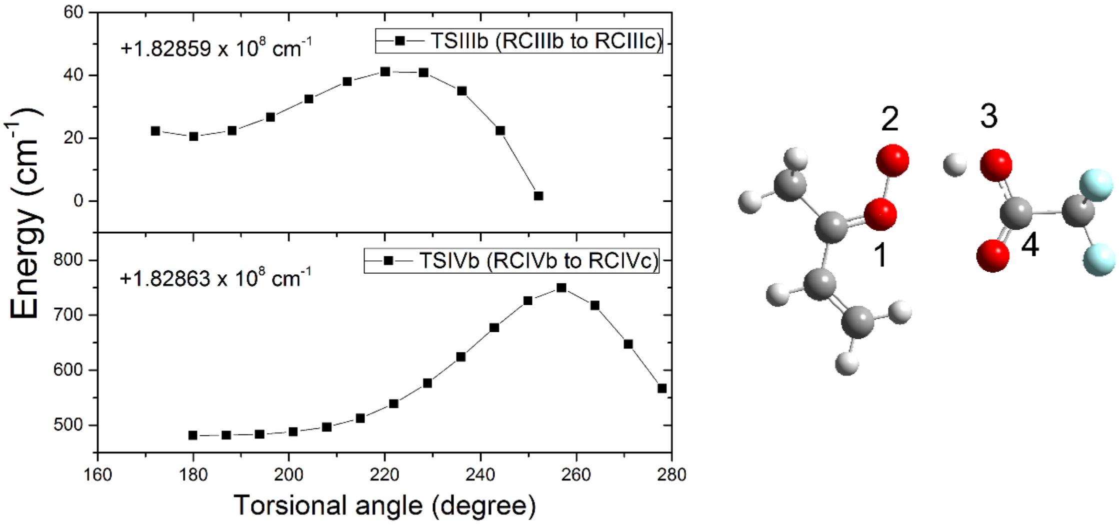

Figure S10: Relaxed scans calculated at the B3LYP/6-31+G(d) level of theory for the torsional step in reactions III and IV of CF_3COOH with anti-trans-C(CH_3)=CH_2)-CHOO and syn-CH_3-trans-(CH=CH_2)COO respectively. *The dihedral angle used for the scan is shown by the labels 1, 2, 3 and 4. The energies were shifted by the amounts labelled in the panels for clarity.*

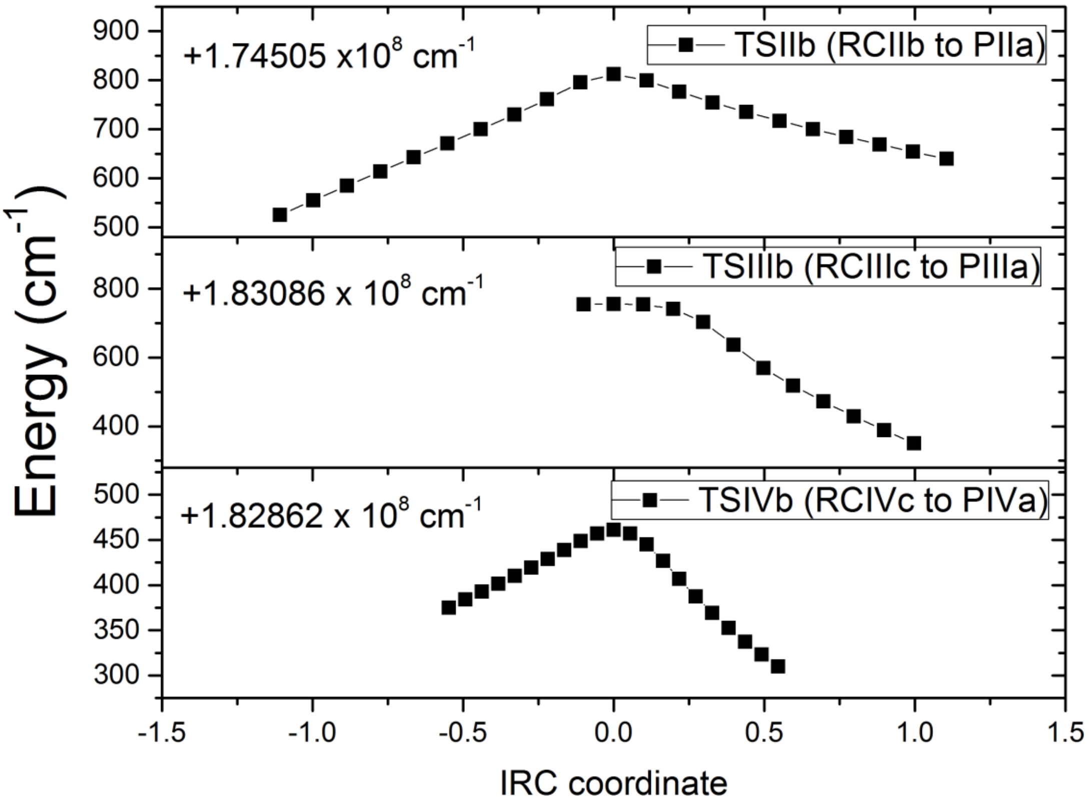

Figure S11: Intrinsic reaction coordinate calculated at the B3LYP/6-31+G(d) level of theory for the H atom transfer step in reactions II, III and IV of CF_3COOH with $(CH_3)_2COO$, anti-trans-C(CH_3)=CH_2)-CHOO and syn-CH_3-trans-(CH=CH_2)COO respectively. The energies were shifted by the amounts labelled in the panels for clarity.

S5 Atmospheric Chemistry Modelling

S5.1 Tropospheric reactions of TFA with Criegee intermediates

The rate coefficient data determined in this study have been used in conjunction with Criegee intermediate fields derived from the STOCHEM-CRI[9] global atmospheric chemistry and transport model to determine loss rates for TFA and to compare these with gas-phase loss rates with the OH radical and depositional loss (wet and dry). The model and the methodology for generating Criegee fields have been described previously.[10] Figure S12 shows the Criegee field generated using ozonolysis reactions of six alkenes (ethene, propene, *trans*-but-2-ene, isoprene, α-pinene and β-pinene) and their loss through unimolecular reaction and reactions with water and water dimer. A lumped unimolecular reaction rate of 100 s^{-1} and rate coefficient of 1×10^{-16} cm^3 s^{-1} for reaction with water were used as the sink reactions for all the Criegee intermediates. Recent work has shown that a rapid reaction exists between CH_2OO and the water dimer $(H_2O)_2$[11] and inclusion of these data in the model suggests that this reaction reduces the steady-state concentration of CH_2OO to low levels. Therefore, the two scenarios presented in this work refer to the impact of all Criegee intermediates excluding CH_2OO, which reduces the model Criegee intermediate fields by approximately 30% from previous estimates, in keeping with estimates by Li *et al*.[12] concerning the percentage level of CH_2OO within the total Criegee intermediate concentration. An average global Criegee intermediate steady state concentration of 3330 molecule cm^{-3} was obtained from the model using this approach.

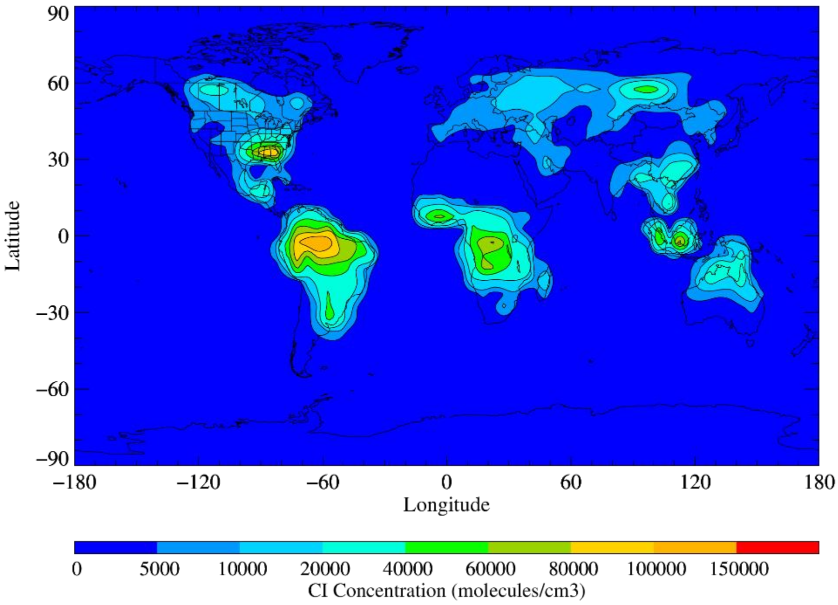

Figure S12: An estimate of annual surface Criegee intermediate concentration derived using the global model, STOCHEM-CRI. The contribution of CH_2OO is excluded because of the rapid reaction of CH_2OO with water dimer.

In scenario A, all Criegee intermediates generated in the model react with the same rate coefficient as $(CH_3)_2COO$ and in scenario B , all Criegee intermediates generated in the model react with the same rate coefficient as CH_2OO. For each scenario, we compare the contribution of Criegee intermediates to the total loss rate for TFA from reaction with OH radicals, depositional loss using parameters stated by Hurley *et al.*,[13] and Criegee intermediates. The outcomes are shown in Fig. S13 (scenario A) and Fig. 4 in the main text (scenario B). Although there are differences between the two scenarios in terms of absolute loss rates, the results are essentially the same: the presence of Criegee intermediates increases the loss of TFA significantly both globally and regionally, and therefore reduces the lifetime of this species. Such a reduction in lifetime suggests sources of atmospheric TFA must be greater than current estimates to balance the budget. The lifetime of TFA is estimated to be 10 days,[13] but based on the model fields and rate coefficients determined in this study, we suggest that the lifetime could be as short as 4 days.

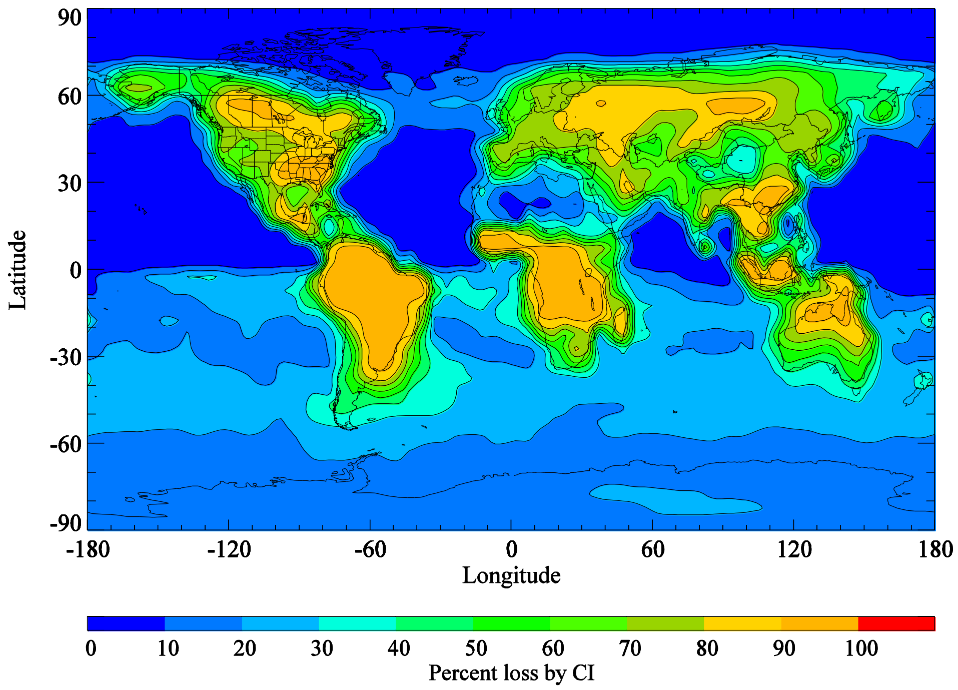

Figure S13: Annual mean CF_3COOH loss contribution by Criegee intermediates (as a percentage of the total loss rate) using $k_{CF3COOH+(CH3)2COO}$ for all Criegee intermediates. Note: Percent loss by CI = (loss by CI $\times$ 100)/(loss by CI + loss by OH + deposition loss).

However, the fates of the products of the various loss processes should be considered. The adduct product has a carbonyl group and may have significant absorption cross section in the actinic region. Absorbed UV photons could cause photodissociation of C-C, O-C or O-O bonds, hence initiating oxidation chemistry.

For the reaction with OH, abstraction dominates (SR1).[13]

$OH + CF_3C(O)OH \rightarrow H_2O + CF_3C(O)O$ (SR1)

$CF_3C(O)O + M \rightarrow CF_3 + CO_2 + M$ (SR2)

$CF_3 + O_2 + M \rightarrow CF_3O_2 + M$ (SR3)

The alkoxy radical, $CF_3C(O)O$ will then decompose through SR2 and form the peroxy radical CF_3O_2 through reaction (SR3). Therefore, TFA is removed but the CF_3 moiety remains in the atmosphere.

Evidence from the electronic structure calculations reported in the main manuscript and here points to an HPE addition reaction of the Criegee intermediate and the TFA.

$CR_1R_2OO + CF_3C(O)OH \rightarrow CF_3C(O)OCR_1R_2OOH$ (SR4)

The absence of a kinetic isotope effect in $CF_3C(O)OD$ and $CF_3C(O)OH$ reactions is consistent with this chemistry. We now consider the fate of the HPE products in the atmosphere. Estimated vapour pressures (see Section S5.2) for the HPE species $CF_3C(O)OCR_1R_2OOH$ of 0.001 atm for R_1=R_2 = H and 0.0001 atm for R_1=R_2 = CH_3 are, respectively, one and two orders of magnitude smaller than the 0.019 atm vapour pressure of $CF_3C(O)OH$. The HPE products will also be less acidic than TFA. Therefore, reaction with Criegee intermediates is proposed to lead to permanent removal of TFA from the atmosphere through deposition.

S5.2 Vapour pressure estimation

Vapour pressure estimates were made using the Nannoolal/Nannoolal method, which was previously identified as one of the best estimation methods for highly functionalised, low volatility compounds.[14] A brief summary is repeated here. The method is based on group contributions and includes 207 primary and secondary groups and group interactions. The first step is to calculate a normal boiling point, T_b for the compound of interest:

$$T_b = \frac{\sum_i N_i C_i}{n^a + b} + c \qquad \text{(S9)}$$

where N_i is the number of groups of type *i*, C_i is the group contribution of group *I*, *n* is the total number of atoms in the molecule (excluding hydrogen), and *a*, *b* and *c* are adjustable parameters from a linear regression of the equation to experimental boiling points. The boiling point estimate is discussed in further detail by Nannoolal *et al.*[15] The boiling point estimate is then used as an input to Eq. (S10), and the vapour pressure is calculated by extrapolating to the temperature of interest:

$$log_{10} P_i^0 = (4.102 + dB)\left[\frac{T - T_b}{T - 0.125 T_b}\right] \qquad \text{(S10)}$$

Here, dB adjusts the slope of the vapour pressure curve and is calculated by a group contribution method:

$$dB = (\sum N_i C_i + GI) - 0.176055 \qquad \text{(S11)}$$

The first term in the brackets is the sum of the primary and secondary group contributions, and the second term is the group interaction:

$$GI = \frac{1}{n}\sum_{i=1}^{m}\sum_{j=1}^{m}\frac{C_{i-j}}{m-1} \qquad \text{(S12)}$$

where $C_{i\text{–}j} = C_{j\text{–}I}$ and m, n are the total number of interacting groups and the number of (non-hydrogen) atoms in the molecule, respectively. The vapour pressure estimate is discussed in further detail by Nannoolal *et al*.[16] The vapour pressures were calculated from SMILE strings generated for each compound and processed on the University of Manchester property estimation calculator site (http://umansysprop.seaes.manchester.ac.uk/tool/vapour_pressure).

References

[1] O. Welz, J. D. Savee, D. L. Osborn, S. S. Vasu, C. J. Percival, D. E. Shallcross, C. A. Taatjes, *Science* **2012**, *335*, 204-207.

[2] R. Chhantyal-Pun, A. Davey, D. E. Shallcross, C. J. Percival, A. J. Orr-Ewing, *Phys. Chem. Chem. Phys.* **2015**, *17*, 3617-3626.

[3] a) W.-L. Ting, Y.-H. Chen, W. Chao, M. C. Smith, J.-M. Lin, *Phys. Chem. Chem. Phys.* **2014**, *16*, 10438-10443; b) R. Chhantyal-Pun, O. Welz, J. D. Savee, A. J. Eskola, E. P. F. Lee, L. Blacker, H. R. Hill, M. Ashcroft, M. A. H. Khan, G. C. Lloyd-Jones, L. Evans, D. Y. Sasaki, B. Rotavera, H. Huang, A. M. Scheer, D. L. Osborn, D. K. W. Mok, J. M. Dyke, D. E. Shallcross, C. J. Percival, A. J. Orr-Ewing, C. A. Taatjes, *J. Phys. Chem. A* **2017**, *121*, 4-15.

[4] a) H.-L. Huang, W. Chao, J.-M. Lin, *Proc. Natl. Acad. USA* **2015**, *112*, 10857-10862; b) L. Sheps, *J. Phys. Chem. Lett* **2013**, *4*, 4201–4205; cR. Dawes, B. Jiang, H. Guo, *J. Am. Chem. Soc.* **2014**, *137*, 50-53.

[5] A. I. Maergoiz, E. E. Nikitin, J. Troe, V. G. Ushakov, *J. Chem. Phys.* **1996**, *105*, 6277.

[6] a) B. Long, J.-R. Cheng, X.-F. Tan, W.-J. Zhang, *J. Mol. Struct. Theochem* **2009**, *916*, 159-167; b) M. Kumar, D. H. Busch, B. Subramaniam, W. H. Thompson, *Phys. Chem. Chem. Phys.* **2014**, *16*, 22968-22973.

[7] M. J. Frisch, G. W. Trucks, H. B. Schlegel, G. E. Scuseria, M. A. Robb, J. R. Cheeseman, G. Scalmani, V. Barone, B. Mennucci, G. A. Petersson, H. Nakatsuji, M. Caricato, X. Li, H. P. Hratchian, A. F. Izmaylov, J. Bloino, G. Zheng, J. L. Sonnenberg, M. Hada, M. Ehara, K. Toyota, R. Fukuda, J. Hasegawa, M. Ishida, T. Nakajima, Y. Honda, O. Kitao, H. Nakai, T. Vreven, J. A. Montgomery, J. E. P. Jr., F. Ogliaro, M. Bearpark, J. J. Heyd, E. Brothers, K. N. Kudin, V. N. Staroverov, R. Kobayashi, J. Normand, K. Raghavachari, A. Rendell, J. C. Burant, S. S. Iyengar, J. Tomasi, M. Cossi, N. Rega, J. M. Millam, M. Klene, J. E. Knox, J. B. Cross, V. Bakken, C. Adamo, J. Jaramillo, R. Gomperts, R. E. Stratmann, O. Yazyev, A. J. Austin, R. Cammi, C. Pomelli, J. W. Ochterski, R. L. Martin, K. Morokuma, V. G. Zakrzewski, G. A. Voth, P. Salvador, J. J. Dannenberg, S. Dapprich, A. D. Daniels, Ö. Farkas, J. B. Foresman, J. V. Ortiz, J. Cioslowski, D. J. Fox, *Gaussian 09, Revision D.01*, Gaussian, Inc., Wallingford CT, **2009**.

[8] H.-J. Werner, P. J. Knowles, G. Knizia, F. R. Manby, M. Schütz, P. Celani, W. Györffy, D. Kats, T. Korona, R. Lindh, A. Mitrushenkov, G. Rauhut, K. R. Shamasundar, T. B. Adler, R. D. Amos, A.

Bernhardsson, A. Berning, D. L. Cooper, M. J. O. Deegan, A. J. Dobbyn, F. Eckert, E. Goll, C. Hampel, A. Hesselmann, G. Hetzer, T. Hrenar, G. Jansen, C. Köppl, Y. Liu, A. W. Lloyd, R. A. Mata, A. J. May, S. J. McNicholas, W. Meyer, M. E. Mura, A. Nicklaß, D. P. O'Neill, P. Palmieri, K. Pflüger, R. Pitzer, M. Reiher, T. Shiozaki, H. Stoll, A. J. Stone, R. Tarroni, T. Thorsteinsson, M. Wang, *MOLPRO, version 2015.1, a package of ab initio programs. see* http://www.molpro.net.

[9] S. R. Utembe, M. C. Cooke, A. T. Archibald, M. E. Jenkin, R. G. Derwent, D. E. Shallcross, *Atmos. Environ.* **2010**, *44*, 1609-1622.

[10] a) C. J. Percival, O. Welz, A. J. Eskola, J. D. Savee, D. L. Osborn, D. O. Topping, D. Lowe, S. R. Utembe, A. Bacak, G. McFiggans, M. C. Cooke, P. Xiao, A. T. Archibald, M. E. Jenkin, R. G. Derwent, I. Riipinen, D. W. K. Mok, E. P. F. Lee, J. M. Dyke, C. A. Taatjes, D. E. Shallcross, *Faraday Discuss* **2013**, *165*, 45-73; b) O. Welz, A. J. Eskola, L. Sheps, B. Rotavera, J. D. Savee, A. M. Scheer, D. L. Osborn, D. Lowe, A. M. Booth, P. Xiao, M. A. H. Khan, C. J. Percival, D. E. Shallcross, C. A. Taatjes, *Angew Chem Int Edit* **2014**, *53*, 4547-4550; c) M. A. H. Khan, M. C. Cooke, S. R. Utembe, P. Xiao, W. C. Morris, R. G. Derwent, A. T. Archibald, M. E. Jenkin, C. J. Percival, D. E. Shallcross, *Atmos. Environ.* **2015**, *110*, 65-74.

[11] W. Chao, J.-T. Hsieh, C.-H. Chang, J.-M. Lin, *Science* **2015**, *347*, 751.

[12] J. Y. Li, Q. Ying, B. Q. Yi, P. Yang, *Atmos. Environ.* **2013**, *79*, 442-447.

[13] M. D. Hurley, M. P. S. Andersen, T. J. Wallington, D. A. Ellis, J. W. Martin, S. A. Mabury, *J. Phys. Chem. A* **2004**, *108*, 615-620.

[14] A. M. Booth, M. H. Barley, D. O. Topping, G. McFiggans, A. Garforth, C. J. Percival, *Atmos. Chem. Phys* **2010**, *10*, 4879-4892.

[15] Y. Nannoolal, J. Rarey, D. Ramjugernath, W. Cordes, *Fluid Phase Equilibr.* **2004**, *226*, 45-63.

[16] Y. Nannoolal, J. Rarey, D. Ramjugernath, *Fluid Phase Equilibr.* **2008**, *269*, 117-133.
